# Molecular characteristics and phylogenetic analysis of pigeon paramyxovirus type 1 isolates from pigeon meat farms in Shanghai (2009–2012)

**DOI:** 10.1038/s41598-024-61235-8

**Published:** 2024-05-10

**Authors:** Wenwei Sheng, Kexuan Wang, Yaping Gui, Xinyong Qi, Liping Shen, Yujie Zhang, Congsheng Tang, Xin Li, Jun Tao, Chuangui Cao, Weidong Qian, Jian Liu

**Affiliations:** 1 Shanghai Animal Disease Prevention and Control Center, Shanghai, 201103 China; 2 School of Life Sciences, Fudan University, Shanghai, 200438 China; 3 Shanghai Jiading District Agricultural Technology Extension Service Center, Shanghai, 201800 China; 4 Shanghai Jiading District Animal Disease Prevention and Control Center, Shanghai, 201800 China; 5 School of Biomedical and Pharmaceutical Sciences, Shaanxi University of Science and Technology, Xi’an, 710021 Shaanxi China

**Keywords:** Paramyxovirus type 1, Phylogeny, Pigeon, Molecular characterization, Genetic variation, Viral evolution

## Abstract

The majority of pigeon paramyxovirus type 1 (PPMV-1) strains are generally non-pathogenic to chickens; however, they can induce severe illness and high mortality rates in pigeons, leading to substantial economic repercussions. The genomes of 11 PPMV-1 isolates from deceased pigeons on meat pigeon farms during passive monitoring from 2009 to 2012 were sequenced and analyzed using polymerase chain reaction and phylogenetic analysis. The complete genome lengths of 11 isolates were approximately 15,192 nucleotides, displaying a consistent gene order of 3′-NP-P-M-F-HN-L-5′. ALL isolates exhibited the characteristic motif of 112RRQKRF117 at the fusion protein cleavage site, which is characteristic of velogenic Newcastle disease virus. Moreover, multiple mutations have been identified within the functional domains of the F and HN proteins, encompassing the fusion peptide, heptad repeat region, transmembrane domains, and neutralizing epitopes. Phylogenetic analysis based on sequences of the F gene unveiled that all isolates clustered within genotype VI in class II. Further classification identified at least two distinct sub-genotypes, with seven isolates classified as sub-genotype VI.2.1.1.2.2, whereas the others were classified as sub-genotype VI.2.1.1.2.1. This study suggests that both sub-genotypes were implicated in severe disease manifestation among meat pigeons, with sub-genotype VI.2.1.1.2.2 displaying an increasing prevalence among Shanghai’s meat pigeon population since 2011. These results emphasize the value of developing pigeon-specific vaccines and molecular diagnostic tools for monitoring and proactively managing potential PPMV-1 outbreaks.

## Introduction

Newcastle disease (ND) stands as a highly contagious viral disease with significant implications for the global poultry industry^1^. Its etiology lies in the Newcastle disease virus (NDV), which is classified under the genus *Orthoavulavirus* within the subfamily *Avulavirinae* of the family *Paramyxoviridae*^2^. NDV comprises an enveloped structure housing a negative-sense, single-stranded RNA genome^3^ spanning approximately 15.2 kb in length. This genome orchestrates the synthesis of six major proteins: nucleocapsid (N), phosphoprotein (P), matrix (M), fusion (F), hemagglutinin-neuraminidase (HN), and large RNA-dependent RNA polymerase (L). Pigeon paramyxovirus type 1 (PPMV-1) emerges as a distinctive antigenically variant genotype within the NDV spectrum, boasting a unique monoclonal antibody binding profile. Originating in the Middle East during the late 1970s, PPMV-1 has since proliferated across Europe and established a global presence^4^.

Phylogenetic analysis of the F gene has delineated PPMV-1 into two discrete classes: class I and class II. According to the current unified phylogenetic classification system, class I encompasses three sub-genotypes: 1.1.1, 1.1.2, and 1.2^5^. Predominantly, class I viruses exhibit avirulent traits and predominantly circulate among wild avian populations. In contrast, Class II demonstrates greater diversity, with identification of at least 20 distinct genotypes (I to XXI, excluding XV)^6^. Among them, genotype VI NDVs, also recognized as PPMV-1 viruses, represent variant strains of NDV commonly associated with infections of Columbiformes, inclusive of both wild and domestic pigeons and doves. Additionally, PPMV-1 is implicated in Newcastle disease outbreaks among chickens, with its virulence potentially escalating following multiple passage^7,8^. Phylogenetic investigations have unveiled genetic diversity among PPMV-1 strains, identifying at least 15 sub-genotypes^9–12^. In accordance with the updated classification criteria for NDV sub-genotypes, PPMV-1 strains have been categorized into six distinct sub-genotypes: VI.1, VI.2.1, VI.2.1.2, VI.2.2.1, VI.2.2.2, VI.2.1.1.1, VI.2.1.1.2.1, and VI.2.1.1.2.2^5^.

Most PPMV-1 strains pose little threat to chickens but can inflict severe illness and mortality in pigeons, leading to considerable economic losses. The principal clinical manifestations in pigeons infected with PPMV-1 include neurological symptoms such as paralysis, torticollis, and abnormal head and neck movements^13,14^. In this study, we scrutinized the genetic characteristics of 11 PPMV-1 isolates isolated from pigeon meat farms in Shanghai between 2009 and 2012. Our analysis unveiled both commonalities and disparities among the 11 PPMV-1 strains at the molecular level, notably observing distinctions in the F and HN proteins between the PPMV-1 isolates and the La Sota strain. These findings hold promise for the development of molecular diagnostic tools and the formulation of vaccines aimed at monitoring or preventing potential PPMV-1 outbreaks.

## Materials and methods

### Sample collection

Between 2009 and 2012, eleven visceral tissue samples, exhibiting evident clinical signs such as neurological and respiratory symptoms, were collected from deceased pigeons that originated from a pigeon meat factory in Shanghai. These samples were promptly stored at − 70 °C. Table S1 provides comprehensive details regarding the collection dates, locations, clinical symptoms, gross lesions, and immune status concerning NDV for each sample. The livers, spleens, lungs, and kidneys of the deceased pigeons underwent homogenization and centrifugation following three rounds of freeze–thaw cycles. The resulting supernatants were then subjected to total RNA extraction.

### Quantitative reverse transcription-polymerase chain reaction (RT-qPCR) for screening positive samples of PPMV-1

Total RNA was extracted from the supernatants using a MagPure Viral RNA Kit (Magen Biotech Co., Ltd., Guangdong, China), following the manufacturer’s guidelines. The primers and probe (forward primer: 5′-GCCATGACTGCRTATGAGAC-3′; reverse primer: 5′-CGGACTGCYAGAGAATGTCTGA-3′; probe: 5′-FAM-CCTRYCATCCYGTATGCAGGAGTGCA-BHQ1-3′) used for screening positive samples of PPMV-1 were synthesized by Saiheng Biotech (Shanghai, China), and conducted in accordance with a previous report^6^. RT-qPCR was conducted in a 20 μL reaction mixture comprising 10 μL of 2× One Step RT-qPCR Buffer (TaKaRa, Dalian, China), 0.4 μL of TaKaRa Ex Taq HS (5 U/μL), 0.4 μL of PrimeScript RT Enzyme Mix, 0.4 μL of forward primer (10 μM), 0.4 μL of reverse primer (10 μM), 0.8 μL of the probe (10 μM), 2 μL of RNA template, and 5.6 μL of RNasefree ddH_2_O. The reactions were conducted using a 7500 real-time PCR instrument (Thermo Fisher Scientific, USA) with the following cycling conditions: 5 min at 42 °C, 10 s at 95 °C, followed by 40 cycles of 5 s at 95 °C and 20 s at 60 °C. Specimens were deemed positive if the cycle threshold value was ≤ 36.0. The comparison results revealed that the RT-qPCR results with a cycle threshold value of ≤ 36.0 cycles were consistent with the Sanger sequencing results, which displayed the corresponding peaks along the nucleotide sequence (data not shown).

### Complete genome sequence assembly of PPMV-1 isolates

The primers used for the complete genome sequence assembly of PPMV-1 isolates in this study were synthesized by Saiheng Biotech and are detailed in Table S2^15^. To perform the RT-PCR reactions, a 50 μL reaction mixture was prepared, consisting of 25 μL of 2 × 1 Step Buffer (Dye Plus), 2 μL of PrimeScript 1 Step Enzyme Mix, 1 μL of forward primer (20 μM), 1 μL of reverse primer (20 μM), 20 μL of RNase-free ddH_2_O, and 1 μL of template RNA. The cycling conditions for RT-PCR were as follows: 50 °C for 30 min, 94 °C for 2 min, followed by 30 cycles of 94 °C for 30 s, 55 °C for 30 s, and 72 °C for 2 min, with a final extension step at 72 °C for 10 min. The resulting RT-PCR products, designated as P1-P10, were subsequently sequenced by Saiheng Biotech. Sequencing data were analyzed using DNAStar software for the complete genome sequence assembly.

### Phylogenetic analysis

The reference strains collected between 1979 and 2016 (Table 1) and the 11 isolates from this study were sourced from NCBI GenBank (https://www.ncbi.nlm.nih.gov/genbank/). The viral sequences of these strains were aligned using MegAlign, a component of the Lasergene package (DNASTAR Inc., Madison, WI, USA). Additionally, phylogenetic analyses of both the full genomes and complete sequences of the F and HN genes were conducted using the neighbor-joining method in MEGA7.0, with 1000 bootstrap replicates. The sub-genotypes and genotypes were designated according to newly proposed classification criteria^5^.

**Table 1:** Information regarding reference isolates used in this study.

Group	Genotype	Isolates	Accession number	Time	Place
Class I		NDV08-004	FJ794269	2008	China
Class I		DE-R49/99	DQ097393	2005	Hungary
Class II	I	98–1252	AY935493	2005	Australia
Class II	I	I-2	AY935499	2005	Australia
Class II	II	La Sota	AF077761	1998	Netherlands
Class II	II	Mallard/US(OH)/86–233/1986	GQ288380	1986	USA
Class II	III	JS/9/05/Go	FJ430160	2008	China
Class II	III	Mukteswar	EF201805	2006	China
Class II	IV	Herts/33	AY741404	2004	Netherlands
Class II	IV	Italien	EU293914	2007	China
Class II	V	Anhinga/U.S.(Fl)/44083/93	AY562986	2004	USA
Class II	V	Gamefowl/U.S.(CA)/211472/02	AY562987	2004	USA
Class II	VII	Chicken/China/Guangxi9/2003	DQ485230	2003	China
Class II	VII	ZJ1	AF431744	2001	China
Class II	VIII	QH1	FJ751918	1979	China
Class II	VIII	QH4	FJ751919	1985	China
Class II	IX	F48E8	FJ436302	2008	China
Class II	IX	JS/1/02/Du	FJ436306	2008	China
Class II	VI.1	GB 1168/84	AF109885	1999	UK
Class II	VI.1	s-1	FJ865434	2002	China
Class II	VI.2.1	PHL37114.2	MH377247	2008	Israel
Class II	VI.2.1	PHL264752	MH377302	2012	Israel
Class II	VI.2.1.2	Pigeon/Nigeria/NIE09-1898/2009	HG326604	2009	Luxembourg
Class II	VI.2.1.2	Pigeon/Nigeria/NIE13-092/2013	HG424627	2013	Luxembourg
Class II	VI.2.1.1.1	APMV1/Pigeon/NJ/USA/0721/2007	JX901351	2007	USA
Class II	VI.2.1.1.1	APMV1/Pigeon/PA/USA/0810/2008	JX901367	2008	USA
Class II	VI.2.1.1.2.1	Pi/CH/LHLJ/110813	JX486553	2011	China
Class II	VI.2.1.1.2.1	sms12	JX094510	2012	China
Class II	VI.2.1.1.2.2	Pigeon/Ningxia/2068/2016	MG840654	2016	China
Class II	VI.2.1.1.2.2	Pi/SH/CH/0167/2013	KT163262	2013	China
Class II	VI.2.2.1	Pigeon/Texas/209682/2002	JN872180	2001	USA
Class II	VI.2.2.2	Pigeon/China/100/08	JX244794	2001	USA
Class II	VI.2.2.2	PG/CH/JS/1/05	FJ480825	2005	China

### Analysis of amino acid mutation sites in the F and HN proteins

The amino acid sequences in the F and HN proteins were aligned to compare the amino acid mutation sites of the classic vaccine strain La Sota with those of the 11 isolates^15^. A comparative analysis was then conducted on the glycosylation sites, cysteine residues, and neutral epitope antigens of the F and HN proteins in each of the 11 strains, with reference to the La Sota strain.

### Ethics approval and consent to participate

The clinical samples collection was approved Shanghai Animal Disease Prevention and Control Center.

## Results

### Characteristics of complete genome sequences and genetic evolution analysis

Ten overlapping fragments covering the entire genome were amplified using RT-PCR to ensure the accuracy of the sequences at both ends of the gene fragments. These fragments were sequenced and subsequently assembled into a single contiguous sequence. The GenBank accession numbers for the full-length genome sequences of the 11 isolates are as follows: Pi/SH/CH/022401/2009, Pi/SH/CH/010502/2010, Pi/SH/CH/010515/2010, Pi/SH/CH/010516/2010, Pi/SH/CH/041002/2011, Pi/SH/CH/061002/2011, Pi/SH/CH/120203/2011, Pi/SH/CH/040601/2012, Pi/SH/CH/050201/2012, Pi/SH/CH/051401/2012, and Pi/SH/CH/051402/2012 were PP297106, PP297105, PP297104, PP297103, PP297102, PP297101, PP297100, PP297099, PP297098, PP297097, and PP297096, respectively. The full-length genome sequence of the 11 PPMV-1 isolates was 15,192 nucleotides in length, organized as 3′-NP-P-M-F-HN-L-5′ (Table 2).

**Table 2:** Genome characteristics of PPMV-1 isolates.

Region	Gene sequence	3′ UTR	Coding sequence^a^	5′ UTR	Intergenic region	Nucleotide length	Amino acid length^b^
3′ Leader	1–55					55	
NP	56–1808	66	122–1591	217	1	1753	489
P	1810–3260	83	1893–3080	180	1	1451	395
M	3262–4502	34	3296–4390	112	1	1241	364
F	4504–6295	46	4550–6211	84	31	1792	553
HN	6327–8328	91	6418–8133	195	47	2002	571
L	8376–15,078	11	8387–15,001	77		6703	2204
5′ Trailer	15,079–15,192					114	

All eleven isolates used in this study showed same genome length characteristics.

^a^including stop codon.

^b^without stop codon.

As shown in Table 2 , the 3′ leader and 5′ trailer lengths of 11 PPMV-1 isolates were 55 and 114 nucleotides, respectively. The intergenic sequence (IGS) lengths for NP-P, P-M, and M-F were 1 nt. The amino acid lengths of NP, P, M, HN, and L proteins were 489, 395, 364, 571, and 2204, respectively, while the F protein length was 553 aa, consistent with NDV type VI F protein. The F protein cleavage site in all isolates was 112RRQKRF117, characteristic of velogenic NDV. Additionally, the IGS lengths for F-HN and HN-L among all 11 PPMV-1 isolates were 31 nt and 47 nt, respectively. Typically, the genes encoding structural proteins contain start and stop codon sequences, and IGS separate each protein gene from one another, ensuring the accuracy of their expression.

The homology analysis of the whole genome sequences showed that the 11 isolates exhibited homologies ranging from 94.7 to 99.9% with Pi/SH/CH/0167/2013, 94.1% to 96.6% with Pi/CH/LHLJ/110813, and 94.0% to 98.6% with pigeon/Ningxia/2068/2016. Furthermore, they also exhibited 85.3% to 85.6% homology with the classical virulent strain F48E8 and 82.9% to 83.2% and 85.6% to 86.0% homology with vaccine strains La Sota and Mukteswar, respectively (Table S3).

### Genetic evolution analysis of F and HN genes

The evaluation of the evolution of the F and HN genes among the 11 isolates revealed a substantial level of homology in both nucleotide and amino acid sequences (Tables S4, S5). Specifically, the nucleotide and amino acid sequences of the F gene demonstrated homology ranging from 95.2 to 100%, while those of the HN gene ranged from 94.8 to 100%. When compared to the La Sota strain, the 11 isolates exhibited nucleotide homologies of 83.7% to 84.8% and amino acid homologies of 88.8% to 89.7% in the F gene. Furthermore, the nucleotide homology of HN with the La Sota strain ranged from 82.9 to 83.7%, while the amino acid homology ranged from 88.1 to 88.6% (Tables S4, S5).

**Figure 1:**
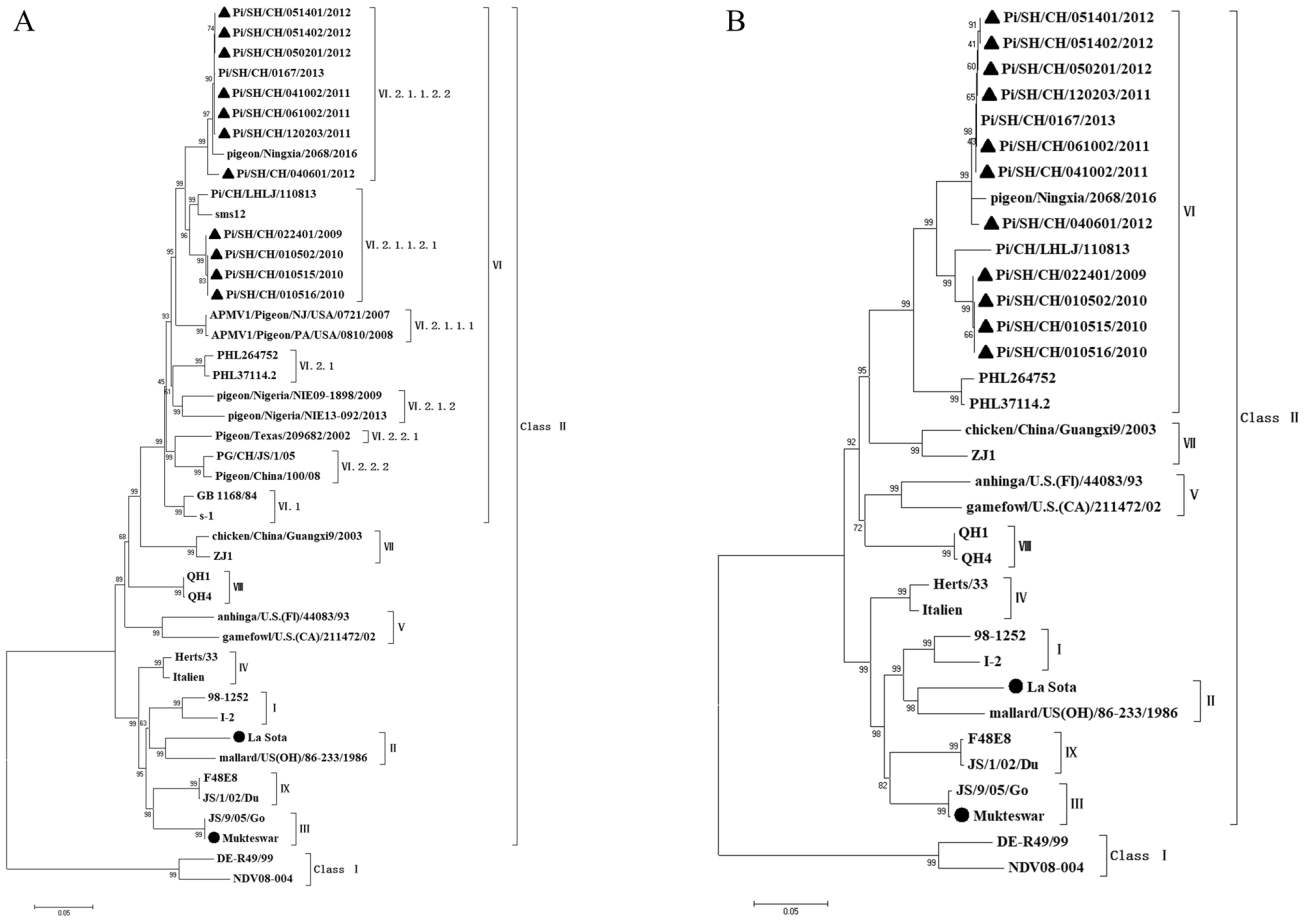
Phylogenetic tree of 11 isolates based on the F (**A**) and HN (**B**) gene sequences is depicted. The sequences of the 11 isolates are denoted by black triangles, while the sequences of the vaccine strains are marked with black circles. Other reference strains were sourced from the National Centre for Biotechnology website. The phylogenetic tree was constructed using MEGA 7.0 software, employing the neighbor-joining method and a 1000 bootstrap analysis. The scale bar represents 0.05.

The phylogenetic analysis of the F and HN genes revealed that the 11 isolates displayed the most distant genetic relationship with the Class I strain DE-R4999 and also showed a distant genetic relationship with the traditional vaccine strain La Sota (Fig. 1A,B). Notably, the 11 isolates belonged to the Class II group of NDV and were classified into either sub-genotype VI.2.1.1.2.1 or VI.2.1.1.2.2. Among them, strains Pi/SH/CH/022401/2009, Pi/SH/CH/010502/2010, Pi/SH/CH/010515/2010, and Pi/SH/CH/010516/2010 were closely associated with strains from sub-genotype VI.2.1.1.2.1, sharing the same evolutionary branch as Pi/CH/LHLJ/110813 and sms12, which were of the same genotype and exhibited the closest genetic relationship (Fig. 1A). In contrast, strains Pi/SH/CH/061002/2011, Pi/SH/CH/120203/2011, Pi/SH/CH/040601/2012, Pi/SH/CH/050201/2012, Pi/SH/CH/051401/2012, and Pi/SH/CH/051402/2012 were classified under sub-genotype VI.2.1.1.2.2, sharing the same evolutionary branch as Pi/SH/CH/0167/2013 and pigeon/Ningxia/2068/2016.

### Analysis of amino acid mutation sites in F and HN genes

The F gene of the 11 isolates was 1792 bp in length, encoding 553 amino acids and containing six potential glycosylation sites (85NRT87, 191NNT193, 366NTS368, 447NVS449, 471NNS473, and 541NNT543). Moreover, 12 cysteine residues were identified at positions 25, 76, 199, 338, 347, 362, 370, 394, 399, 401, 424, and 523 of the F protein in isolates Pi/SH/CH/022401/2009, Pi/SH/CH/010502/2010, Pi/SH/CH/010515/2010, and Pi/SH/CH/010516/2010, with an additional cysteine residue at position 27 in the other seven isolates. The F protein’s transmembrane region exhibited variability, with two substitutions (I502V, V509I) in the first group of isolates and two substitutions (V506I, V509I) in the remaining seven isolates (Table 3). When compared to consensus amino acid sequences from NDV strains of various genotypes, the 11 isolates showed two substitutions (V121I and A132S) in the fusion peptide. Analysis of the three heptad repeat regions (HR) revealed one substitution (V179I) in HRa (143–185 aa) in Pi/SH/CH/061002/2011, Pi/SH/CH/120203/2011, Pi/SH/CH/040601/2012, Pi/SH/CH/050201/2012, Pi/SH/CH/051401/2012, and Pi/SH/CH/051402/2012 isolates (Table 3).

**Table 3:** Amino acid substitutions in the functional domains of F and HN proteins.

Isolate name	F protein					HN protein
	Fusion peptide 117–141	HRa 143–185	HRb 268–299	HRc 471–500	Transmembrane domain 501–521	Transmembrane domain 25–45
Consensus sequence^a^	FIGAVIGSVALGVATAAQITAAAAL	QANQNAANILRLKESIAATNEAVHEVTDGLSQLAVAVGKMQQF	LITGNPILYDSQTQLLGIQVNLPSVGNLNNMR	NNSISNALDKLAESNSKLDKVNVKLTSTSA	LITYIVLTVISLVFGALSLVL	FRIAVLLLIVMTLAISAAALV
Pi/SH/CH/022401/2009	V121I A132S	–	–	–	I502V–V509I	V34A M35V A41V
Pi/SH/CH/010502/2010	V121I A132S	–	–	–	I502V–V509I	V34A M35V A41V
Pi/SH/CH/010515/2010	V121I A132S	–	–	–	I502V–V509I	V34A M35V A41V
Pi/SH/CH/010516/2010	V121I A132S	–	–	–	I502V–V509I	-M35V A41V
Pi/SH/CH/041002/2011	V121I A132S	-V179I	–	–	-V506I V509I	-M35V A41V
Pi/SH/CH/061002/2011	V121I A132S	-V179I	–	–	-V506I V509I	-M35V A41V
Pi/SH/CH/120203/2011	V121I A132S	-V179I	–	–	-V506I V509I	-M35V A41V
Pi/SH/CH/040601/2012	V121I A132S	-V179I	–	–	-V506I V509I	-M35V A41V
Pi/SH/CH/050201/2012	V121I A132S	-V179I	–	–	-V506I V509I	-M35V A41V
Pi/SH/CH/051401/2012	V121I A132S	-V179I	–	–	-V506I V509I	-M35V A41V
Pi/SH/CH/051402/2012	V121I A132S	-V179I	–	–	-V506I V509I	-M35V A41V

^a^The consensus amino acid sequence was derived from NDV strains of different genotypes.

**Figure 2:**
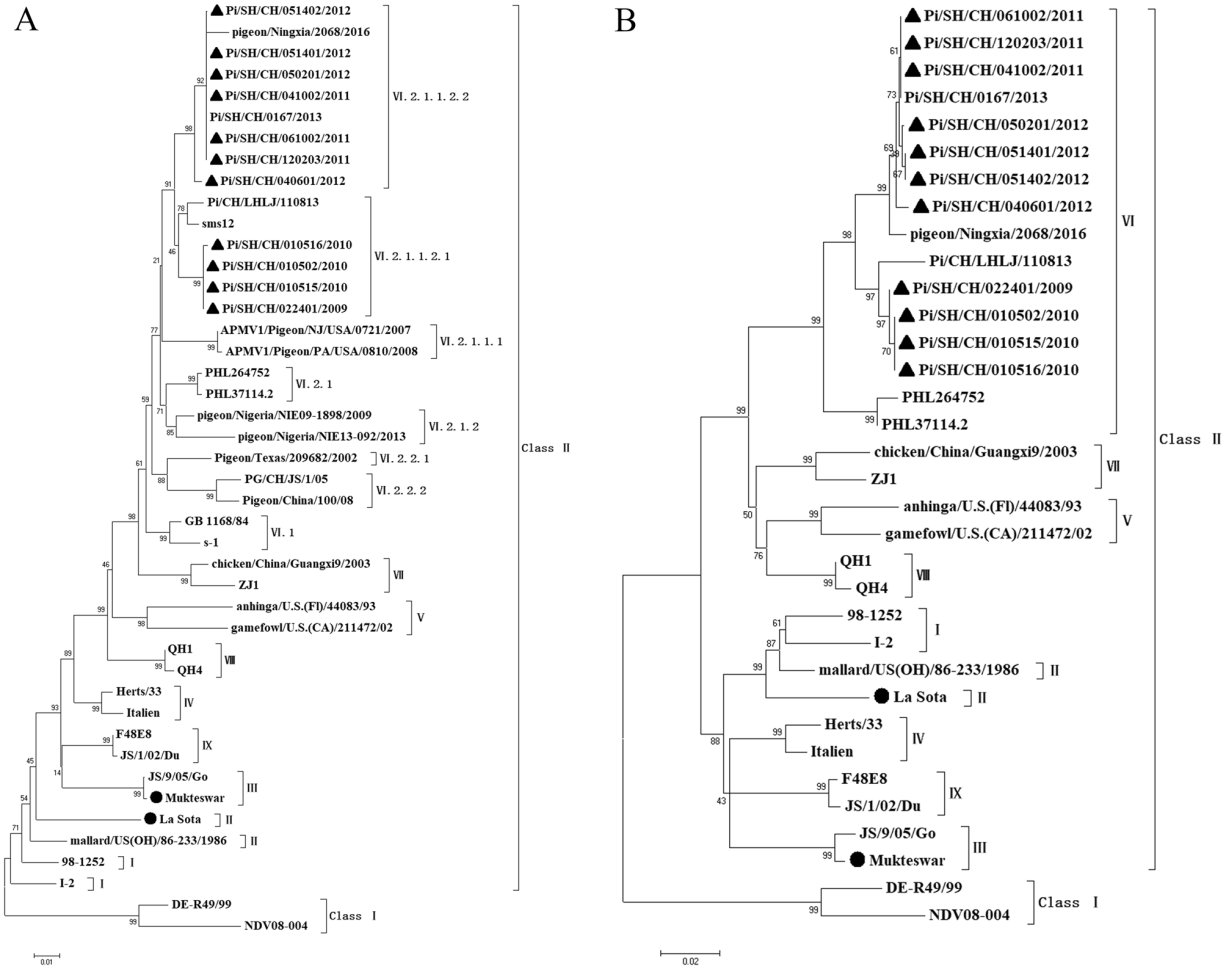
The phylogenetic tree of 11 isolates based on the F (**A**) and HN (**B**) amino acid sequences is illustrated. The sequences of the 11 isolates are represented by black triangles, while the sequences of the vaccine strains are indicated by black circles. Reference strains from the National Center for Biotechnology website were also included. The phylogenetic tree was constructed using MEGA 7.0 software, utilizing the neighbor-joining method and a 1000 bootstrap analysis. The scale bar represents 0.01 (**A**) and 0.02 (**B**), respectively.

The HN gene of the 11 isolates spanned 2002 nucleotides, encoding 571 amino acids, with five potential glycosylation sites (119NNS121, 341NNT343, 433NKT435, 481NHT483, and 508NIS510). Thirteen cysteine residues were situated at positions 123, 172, 186, 196, 238, 247, 251, 344, 455, 461, 465, 531, and 542. The transmembrane region of the HN protein in the 11 isolates exhibited variability, with three substitutions (V34A, M35V, and A41V) in the first group of isolates and two substitutions (M35V and A41V) in the remaining seven isolates (Table 3). Examination of the neutralizing epitopes in the HN protein revealed six amino acid substitutions in Pi/SH/CH/022401/2009, Pi/SH/CH/010502/2010, Pi/SH/CH/010515/2010, and Pi/SH/CH/010516/2010, and seven substitutions in the other seven isolates (Table 4). Furthermore, the phylogenetic analysis of the F and HN amino acids revealed that the 11 isolates showed a distant genetic relationship with the conventional vaccine strain LaSota, which was consistent with the nucleotide acid alignments (Fig. 2A,B).

**Table 4:** Amino acids sequences constituting the neutralizing epitopes of HN protein.

Virus	HN protein									
	193–201	263	287	321	332–333	346–353	356	494	513–521	569
Vaccine strains^a^	LSGCRDHSH	N	D	K	GK	DEQDYQIR	K	G	RITRVSSSS	D
Pi/SH/CH/022401/2009	R197I	K	–	–	–	E347G D349E	–	D	I514V	D
Pi/SH/CH/010502/2010	R197I	K	–	–	–	E347G D349E	–	D	I514V	D
Pi/SH/CH/010515/2010	R197I	K	–	–	–	E347G D349E	–	D	I514V	D
Pi/SH/CH/010516/2010	R197I	K	–	–	–	E347G D349E	–	D	I514V	D
Pi/SH/CH/041002/2011	R197I	K	–	–	–	E347G D349E	–	D	I514V	E
Pi/SH/CH/061002/2011	R197I	K	–	–	–	E347G D349E	–	D	I514V	E
Pi/SH/CH/120203/2011	R197I	K	–	–	–	E347G D349E	–	D	I514V	E
Pi/SH/CH/040601/2012	R197I	K	–	–	–	E347G D349E	–	D	I514V	E
Pi/SH/CH/050201/2012	R197I	K	–	–	–	E347G D349E	–	D	I514V	E
Pi/SH/CH/051401/2012	R197I	K	–	–	–	E347G D349E	–	D	I514V	E
Pi/SH/CH/051402/2012	R197I	K	–	–	–	E347G D349E	–	D	I514V	E

^a^The consensus amino acid sequence of the La Sota vaccine strain.

## Discussion

Since its first identification in China in 1985^16^, PPMV-1 has persisted in pigeon populations despite vaccination efforts, including the use of LaSota vaccine, leading to significant economic losses^17^. Genotype VI encompasses a diverse range of viral strains and has been further subdivided into multiple sub-genotypes^5^. Guo et al.^17^ isolated eight PPMV-1 strains from four Chinese provinces between 2010 and 2012, all of which were classified as sub-genotype VI.2.1.1.2.2. Similarly, Wang et al.^18^ isolated six strains of PPMV-1 from six provinces in China between 2011 and 2013, with five strains as sub-genotype VI.2.1.1.2.2 and one strain as sub-genotype VI.2.1.1.2.1. Zhan et al.^19^ also isolated 21 strains of pigeon NDV in China between 2007 and 2019, with 18 strains identified as sub-genotype VI.2.1.1.2.2 and three strains as sub-genotype VI.2.1.1.2.1. Additionally, from 2014 to 2021, Yu et al.^20^ isolated 28 strains of PPMV-1 from different regions in China, all classified as sub-genotype VI.2.1.1.2.2. It is noteworthy that a report in 2023 revealed the isolation of PPMV-1 from the patient’s sputum and from cloacal smear samples collected from domesticated pigeons belonging to the patient's neighbor, emphasizing the heightened risk of severe PPMV-1 infection in human patients^21^. These results indicate that PPMV-1 strains originating from pigeons in China predominantly belong to sub-genotype VI.2.1.1.2.2, with certain regional differences observed.

This study conducted the sequencing and analysis of 11 PPMV-1 strains isolated from deceased meat pigeons in Shanghai between 2009 and 2012. Phylogenetic analysis based on the complete F gene indicated that four isolates from 2009 to 2010 belonged to sub-genotype VI.2.1.1.2.1, while seven isolates from 2011 to 2012 were classified as sub-genotype VI.2.1.1.2.2, following the updated unified classification system proposed by Dimitrov et al. in 2019^5^. These results suggest that sub-genotype VI.2.1.1.2.2 has been the predominant strain in meat pigeon populations in Shanghai from 2009 and 2012, which, as discussed above, is similar to the recent trends in China over the past few years. However, additional comprehensive research is required to investigate the viral strain prevalence of PPMV-1 in Shanghai post-2012. Moreover, both sub-genotypes VI.2.1.1.2.1 and VI.2.1.1.2.2 have been linked to notable morbidity and mortality rates in meat pigeons in Shanghai.

Previous research has highlighted the significance of amino acid residues at the F protein cleavage site in determining pathogenicity^22,23^. The motif typically observed at the F protein cleavage site of PPMV-1 is either 112GRQKRF117, identified in isolates from the 1980s, or 112RRQKRF117, present in viruses from later periods^24^. In this study, the 11 isolates exhibited the 112RRQKRF117 motif at the F protein cleavage site, consistent with strains isolated post-1980s and resembling velogenic (highly virulent) NDV strains. Furthermore, the length of the HN protein in these isolates ranged from 571 to 616 amino acids, a characteristic associated with viral genotype^25^. However, the 11 isolates in our study had an HN protein length of 571 amino acids, a feature commonly observed in the most virulent NDV strains^25,26^. Furthermore, the isolated strains showed no variation in glycosylation sites and cysteine residues in the F protein; however, compared to the La Sota strain, the HN protein exhibited the absence of glycosylation sites at positions 538–540 and the presence of additional glycosylation sites at positions 508–510. Additionally, the HN protein had an extra cysteine residue at position 123. Mutations in cysteine residues may play a crucial role in the emergence of antigenic sites during the maturation of the HN protein and in maintaining the structural framework of the F protein.

In the 11 PPMV-1 isolates, compared to consensus sequences from different NDV genotypes, multiple amino acid substitutions were detected in the functional domains of the F and HN proteins. These substitutions, found in the fusion peptide and heptad repeat regions of the F protein, as well as alterations in the transmembrane domain, may impact the fusion activity of the F protein^27^. Likewise, the HN protein exhibited six to seven substitutions in the neutralizing epitopes, including N263K, E347G, and I514V, which are also observed in other NDV isolates^27^. Such substitutions in neutralizing epitopes could give rise to neutralizing escape variants, as these epitopes are crucial for antigenic epitope formation^28–30^. Variations in antigenic determinants, especially those vital for viral attachment and fusion with host cells, can lead to the emergence of escape variants and subsequent vaccine failure^15,27,28^. Hence, the investigation of variant presence is crucial in understanding and addressing these challenges.

PPMV-1 is a prevalent zoonotic pathogen that circulates within the pigeon reservoir and has the potential to sporadically transmit to humans^21^. To reduce potential transmission risks, the comprehensive One Health strategy aims to prevent and control zoonotic pathogens by addressing both the natural environment and the human-built environment. In addition to minimizing the risk of spillover or transmission to humans through limited animal contact, conducting intensive surveillance and retrospective analysis is vital for investigating genetic variability and understanding potential implications for transmission in both wild and domesticated species. Furthermore, utilizing epidemiological data to identify candidate vaccine strains is essential for vaccine development, while identifying the potential causes of infectious disease outbreaks remains paramount. Implementing universal or targeted vaccination strategies based on this data can effectively manage infection and transmission within animal populations as well as between animals and humans.

## Conclusion

In this study, we conducted a thorough genomic analysis of 11 PPMV-1 isolates obtained from deceased meat pigeons in Shanghai between 2009 and 2012. Our results reveal that both VI.2.1.1.2.1 and VI.2.1.1.2.2 strains can cause severe diseases in pigeons, with VI.2.1.1.2.2 becoming increasingly prevalent among meat pigeons in Shanghai since 2011. Furthermore, we identified several substitutions in the functional domains of the F and HN proteins when comparing the PPMV-1 isolates to the La Sota strain. These findings highlight the potential benefits of utilizing pigeon-specific vaccines and molecular diagnostic tools to prevent and proactively manage potential PPMV-1 outbreaks.

### Supplementary Information

Supplementary Tables.

## Data Availability

The datasets generated during the current study are available in the GenBank repository, [https://www.ncbi.nlm.nih.gov/nuccore/PP297106, https://www.ncbi.nlm.nih.gov/nuccore/PP297105, https://www.ncbi.nlm.nih.gov/nuccore/PP297104, https://www.ncbi.nlm.nih.gov/nuccore/PP297103, https://www.ncbi.nlm.nih.gov/nuccore/PP297102, https://www.ncbi.nlm.nih.gov/nuccore/PP297101, https://www.ncbi.nlm.nih.gov/nuccore/PP297100, https://www.ncbi.nlm.nih.gov/nuccore/PP297099, https://www.ncbi.nlm.nih.gov/nuccore/PP297098, https://www.ncbi.nlm.nih.gov/nuccore/PP297097, https://www.ncbi.nlm.nih.gov/nuccore/PP297096].

## Abbreviations

PPMV-1: Pigeon paramyxovirus type 1
ND: Newcastle disease
NDV: Newcastle disease virus
N: Nucleocapsid
P: Phosphoprotein
M: Matrix
F: Fusion
HN: Hemagglutinin-neuraminidase
L: Large RNA-dependent RNA polymerase
RT-PCR: Reverse transcription-polymerase chain reaction
IGS: The intergenic sequence
aa: Amino acids
