# Supplementary material for: Molecular characteristics and phylogenetic analysis of pigeon paramyxovirus type 1 isolates from pigeon meat farms in Shanghai (2009–2012)
Source: Sci Rep. 2024 May 10;14:10741. doi: 10.1038/s41598-024-61235-8 (PMC11087573; doi:10.1038/s41598-024-61235-8)
Supplement: Supplementary file 1 — Supplementary Tables. [file 41598_2024_61235_MOESM1_ESM.doc]

Table Supplementary materials

**Table S1**. Information regarding the samples utilized for isolating PPMV-1 strains

| Isolates | Date | Place | Clinical Signs | Gross Lesions | Immunity (NDV) |
| --- | --- | --- | --- | --- | --- |
| Pi/SH/CH/022401/2009 | 2009.02.24 | Baihe Town, Qingpu District | Fever, emaciated | Gastrointestinal bleeding and necrotic lesions on liver | Not learned |
| Pi/SH/CH/010502/2010 | 2010.01.05 | Zhuqiao Town, Jiading District | Diarrhea, neurological symptoms | Gastrointestinal bleeding, necrotic lesions on liver and pancreas | Not learned |
| Pi/SH/CH/010515/2010 | 2010.01.05 | Zhuqiao Town, Jiading District | Diarrhea, neurological symptoms | Gastrointestinal bleeding, necrotic lesions on liver and pancreas | Not learned |
| Pi/SH/CH/010516/2010 | 2010.01.05 | Zhuqiao Town, Jiading District | Diarrhea, neurological symptoms | Gastrointestinal bleeding, necrotic lesions on liver and pancreas | Not learned |
| Pi/SH/CH/041002/2011 | 2011.04.10 | Pudong New Area | Dead, diarrhea, neurological symptoms | Myocardial, pulmonary, intestinal, and glandular gastric bleeding | Immunized |
| Pi/SH/CH/061002/2011 | 2011.06.10 | Jiading District | Not learned | Not learned | Not learned |
| Pi/SH/CH/120203/2011 | 2011.12.02 | Zhuqiao Town, Jiading District | Diarrhea, twisted neck | Gland stomach and meningeal bleeding | Not immunized |
| Pi/SH/CH/040601/2012 | 2012.04.06 | Xinhe Town, Chongming District | Dead, diarrhea, twisted neck | Liver, kidney and pancreatic swelling, intestinal bleeding and cerebral hemorrhage | Not immunized |
| Pi/SH/CH/050201/2012 | 2012.05.02 | Zhuqiao Town, Jiading District | Diarrhea, twisted neck | Gland stomach, muscular stomach, and duodenal bleeding | Immunized |
| Pi/SH/CH/051401/2012 | 2012.05.14 | Zhuqiao Town, Jiading District | Diarrhea, twisted neck | Gland stomach, muscular stomach, and duodenal bleeding | Immunized |
| Pi/SH/CH/051402/2012 | 2012.05.14 | Zhuqiao Town, Jiading District | Diarrhea, twisted neck | Gland stomach, muscular stomach, and duodenal bleeding | Immunized |

**Table S2**. Primer pairs for the whole genome sequencing of PPMV-1 isolates

| Primer pairs | Primer sequence (5’-3’) | Position in the genome |
| --- | --- | --- |
| F1 | ACCAAACAGAGAATCTGTGAGTTACG | 1-26 |
| R1 | CCGAGCCTCCAGAATGATGTA | 1,189-1,209 |
| F2 | GCATCAGTCTTGGATAAGGGAAC | 1,133-1,155 |
| R2 | CAATGACAGTTCCACTGGTCTCA | 1,931-1,953 |
| F3 | CAACCCAATCCACCAACGA | 1,759-1,777 |
| R3 | GCAGCATCAAAGTGCAGCC | 3,318-3,336 |
| F4 | CGAAGAGATCAGGAAGGTCAAG | 3,038-3,059 |
| R4 | TTATCTCCTGTTACCACAATCCC | 4,670-4,692 |
| F5 | CGAATCATCACGACACCAG | 4,426-4,444 |
| R5 | GCCTCTCCGACCGTTCT | 6,333-6,349 |
| F6 | CAGATGAGAGCCACTACAAGAACA | 6,185-6,208 |
| R6 | TCCTTGGTGTTGCGAGAGATA | 8,196-8,216 |
| F7 | AGCACCAAGGCAGCATACAC | 7,978-7,997 |
| R7 | ATTGAAAGGGTCTCCTACAAACAT | 10,433-10,456 |
| F8 | GAAGAGTTGCCACTTTTATCACG | 10,281-10,303 |
| R8 | CGGATGATGCCCTTAGTGC | 12,038-12,056 |
| F9 | CAAAATGTGACAGCGGAGATG | 11,859-11,879 |
| R9 | AATGGCATCTCTGTCCTCTCG | 13,379-13,399 |
| F10 | AGCGGAAGAGAAATGCTCAGTA | 13,216-13,237 |
| R10 | ACCAAACAAAGATTTGGTGAATG | 15,170-15,192 |
| 3’ outer | CGGAGACAGAATACCGCAAA | 272-291 |
| 3’ inner | AGTGAATACTGGGACCTCAACCT | 216-238 |
| 5’ outer | TTAATATGCTTGACTCGTGCTCA | 14,912-14,934 |
| 5’ inner | CCATAGGTAATGCAGCCAAGG | 14,955-14,975 |

**Table S3**.The nucleotide sequence homology and amino acid homology comparisons of full-length genome of the eleven strains with reference strains (%)

|  | **1** | **2** | **3** | **4** | **5** | **6** | **7** | **8** | **9** | **10** | **11** | **12** | **13** | **14** | **15** | **16** | **17** | **18** | **19** | **20** | **21** | **22** | **23** | **24** | **25** | **26** | **27** | **28** | **29** | **30** |  |
| --- | --- | --- | --- | --- | --- | --- | --- | --- | --- | --- | --- | --- | --- | --- | --- | --- | --- | --- | --- | --- | --- | --- | --- | --- | --- | --- | --- | --- | --- | --- | --- |
| **1** |  | **69.2** | **68.6** | **77.8** | **67.1** | **78.0** | **88.1** | **78.7** | **77.4** | **78.2** | **75.5** | **78.4** | **67.6** | **68.0** | **68.5** | **68.5** | **68.5** | **68.0** | **68.7** | **67.6** | **68.0** | **67.8** | **67.8** | **67.7** | **68.0** | **68.0** | **67.2** | **71.3** | **71.2** | **68.7** | **98-1252** |
| **2** | **85.0** |  | **73.1** | **72.0** | **81.5** | **75.5** | **68.0** | **76.1** | **71.8** | **72.1** | **66.5** | **72.2** | **72.0** | **72.4** | **72.5** | **72.6** | **72.5** | **71.9** | **72.6** | **71.2** | **71.9** | **71.8** | **71.8** | **71.8** | **71.9** | **71.8** | **71.1** | **76.6** | **76.5** | **73.1** | **anhinga/U.S.(Fl)/44083/93** |
| **3** | **84.9** | **86.8** |  | **70.7** | **71.2** | **74.2** | **67.3** | **74.8** | **70.5** | **71.0** | **65.7** | **71.2** | **73.9** | **74.4** | **74.8** | **74.8** | **74.7** | **74.5** | **75.0** | **74.0** | **74.6** | **74.4** | **74.3** | **74.3** | **74.5** | **74.4** | **73.7** | **75.4** | **75.3** | **94.9** | **chicken/China/Guangxi9/2003** |
| **4** | **89.5** | **86.4** | **85.8** |  | **70.1** | **82.2** | **75.3** | **82.9** | **99.2** | **82.4** | **73.7** | **82.5** | **69.4** | **70.1** | **70.3** | **70.3** | **70.3** | **70.0** | **70.5** | **69.4** | **69.9** | **69.8** | **69.7** | **69.7** | **69.9** | **69.9** | **69.1** | **73.9** | **73.8** | **70.7** | **F48E8** |
| **5** | **84.4** | **91.2** | **86.1** | **85.7** |  | **73.5** | **66.2** | **73.8** | **69.8** | **70.2** | **65.2** | **70.3** | **70.5** | **70.3** | **71.2** | **71.2** | **71.1** | **70.8** | **71.2** | **70.1** | **70.8** | **70.7** | **70.6** | **70.6** | **70.8** | **70.7** | **70.0** | **74.8** | **74.7** | **71.4** | **gamefowl/U.S.(CA)/211472/02** |
| **6** | **89.6** | **88.2** | **87.8** | **91.6** | **87.5** |  | **75.8** | **96.4** | **81.9** | **82.7** | **74.3** | **82.8** | **72.5** | **73.5** | **74.1** | **74.1** | **74.1** | **73.8** | **74.2** | **73.0** | **73.8** | **73.6** | **73.6** | **73.6** | **73.8** | **73.6** | **72.7** | **78.3** | **78.2** | **74.5** | **Herts/33** |
| **7** | **94.4** | **84.3** | **84.3** | **88.2** | **83.7** | **88.5** |  | **76.6** | **75.1** | **75.9** | **73.5** | **76.1** | **66.7** | **66.8** | **67.2** | **67.2** | **67.2** | **67.0** | **67.4** | **66.6** | **67.1** | **66.9** | **66.9** | **66.9** | **67.0** | **67.1** | **66.5** | **69.7** | **69.6** | **67.3** | **I-2** |
| **8** | **90.0** | **88.5** | **88.0** | **91.9** | **87.7** | **98.3** | **89.0** |  | **82.6** | **83.4** | **74.7** | **83.5** | **73.1** | **73.8** | **74.4** | **74.4** | **74.4** | **74.2** | **74.5** | **73.5** | **74.1** | **73.9** | **73.9** | **73.8** | **74.1** | **74.0** | **73.3** | **78.8** | **78.7** | **75.0** | **Italien** |
| **9** | **89.4** | **86.3** | **85.7** | **99.7** | **85.6** | **91.4** | **88.1** | **91.8** |  | **82.1** | **73.5** | **82.2** | **69.2** | **69.9** | **70.1** | **70.0** | **70.0** | **69.7** | **70.3** | **69.2** | **69.7** | **69.6** | **69.5** | **69.5** | **69.7** | **69.6** | **68.8** | **73.6** | **73.5** | **70.5** | **JS/1/02/Du** |
| **10** | **89.7** | **86.2** | **86.1** | **91.6** | **85.5** | **91.7** | **88.6** | **92.1** | **91.5** |  | **73.7** | **99.3** | **69.6** | **70.4** | **71.0** | **71.0** | **71.0** | **70.5** | **71.1** | **70.1** | **70.6** | **70.4** | **70.3** | **70.3** | **70.5** | **70.5** | **69.8** | **75.1** | **75.0** | **71.0** | **JS/9/05/Go** |
| **11** | **88.8** | **83.5** | **83.3** | **87.8** | **83.0** | **87.7** | **87.6** | **87.9** | **87.7** | **87.5** |  | **73.9** | **65.1** | **65.4** | **65.8** | **65.9** | **65.8** | **65.6** | **65.9** | **65.0** | **65.5** | **65.4** | **65.4** | **65.4** | **65.5** | **65.4** | **64.8** | **67.7** | **67.7** | **65.9** | **LaSota** |
| **12** | **89.8** | **86.3** | **86.2** | **91.7** | **85.6** | **91.7** | **88.7** | **92.1** | **91.6** | **99.7** | **87.6** |  | **69.7** | **70.6** | **71.1** | **71.1** | **71.1** | **70.7** | **71.2** | **70.3** | **70.7** | **70.6** | **70.5** | **70.5** | **70.7** | **70.7** | **69.9** | **75.1** | **75.0** | **71.1** | **Mukteswar** |
| **13** | **84.2** | **86.4** | **87.4** | **85.1** | **85.8** | **86.9** | **83.7** | **87.3** | **85.0** | **85.2** | **82.8** | **85.3** |  | **83.3** | **83.4** | **83.4** | **83.4** | **83.0** | **83.6** | **82.5** | **83.0** | **82.8** | **82.8** | **82.8** | **83.0** | **83.0** | **81.6** | **73.8** | **73.7** | **74.0** | **PHL264752** |
| **14** | **84.4** | **86.4** | **87.5** | **85.4** | **85.6** | **87.2** | **83.8** | **87.5** | **85.3** | **85.7** | **82.9** | **85.8** | **92.1** |  | **92.6** | **92.6** | **92.6** | **88.1** | **92.8** | **87.5** | **88.1** | **87.6** | **87.6** | **87.6** | **88.0** | **87.9** | **86.5** | **74.4** | **74.2** | **74.8** | **Pi/CH/LHLJ/110813** |
| **15** | **84.7** | **86.6** | **87.8** | **85.5** | **86.0** | **87.5** | **84.0** | **87.8** | **85.4** | **85.9** | **83.1** | **85.9** | **92.2** | **96.5** |  | **99.9** | **99.9** | **88.7** | **99.1** | **88.1** | **88.8** | **88.3** | **88.2** | **88.2** | **88.7** | **88.5** | **87.3** | **75.1** | **75.1** | **75.0** | **Pi/SH/CH/010502/2010** |
| **16** | **84.7** | **86.6** | **87.8** | **85.5** | **86.1** | **87.5** | **84.0** | **87.7** | **85.4** | **85.9** | **83.2** | **85.9** | **92.2** | **96.5** | **99.9** |  | **99.8** | **88.6** | **99.1** | **88.1** | **88.7** | **88.2** | **88.2** | **88.2** | **88.6** | **88.5** | **87.3** | **75.0** | **75.0** | **74.9** | **Pi/SH/CH/010515/2010** |
| **17** | **84.7** | **86.5** | **87.8** | **85.5** | **86.0** | **87.5** | **84.0** | **87.8** | **85.4** | **85.9** | **83.1** | **85.9** | **92.2** | **96.5** | **99.9** | **99.9** |  | **88.6** | **99.2** | **88.0** | **88.7** | **88.2** | **88.2** | **88.2** | **88.6** | **88.5** | **87.3** | **75.1** | **75.0** | **74.9** | **Pi/SH/CH/010516/2010** |
| **18** | **84.5** | **86.4** | **87.6** | **85.5** | **85.9** | **87.4** | **84.0** | **87.7** | **85.4** | **85.8** | **83.1** | **85.8** | **92.1** | **94.5** | **94.7** | **94.7** | **94.7** |  | **89.0** | **97.3** | **99.7** | **99.2** | **99.2** | **99.2** | **99.8** | **99.5** | **96.8** | **74.9** | **74.8** | **74.8** | **Pi/SH/CH/0167/2013** |
| **19** | **84.8** | **86.6** | **87.8** | **85.6** | **86.1** | **87.6** | **84.1** | **87.8** | **85.5** | **86.0** | **83.2** | **86.0** | **92.2** | **96.6** | **99.7** | **99.7** | **99.7** | **94.8** |  | **88.4** | **89.1** | **88.6** | **88.5** | **88.5** | **89.0** | **88.8** | **87.5** | **75.2** | **75.2** | **75.2** | **Pi/SH/CH/022401/2009** |
| **20** | **84.3** | **86.1** | **87.4** | **85.3** | **85.6** | **87.1** | **83.8** | **87.4** | **85.2** | **85.6** | **82.9** | **85.6** | **91.8** | **94.1** | **94.4** | **94.4** | **94.4** | **98.7** | **94.6** |  | **97.5** | **96.9** | **96.9** | **96.9** | **97.4** | **97.3** | **95.4** | **74.4** | **74.3** | **74.4** | **Pi/SH/CH/040601/2012** |
| **21** | **84.5** | **86.5** | **87.7** | **85.5** | **85.9** | **87.4** | **84.0** | **87.7** | **85.4** | **85.8** | **83.1** | **85.9** | **92.1** | **94.5** | **94.8** | **94.8** | **94.8** | **99.9** | **94.9** | **98.8** |  | **99.4** | **99.3** | **99.3** | **99.9** | **99.7** | **97.0** | **75.0** | **74.9** | **75.0** | **Pi/SH/CH/041002/2011** |
| **22** | **84.4** | **86.3** | **87.5** | **85.4** | **85.8** | **87.4** | **83.9** | **87.6** | **85.3** | **85.7** | **83.0** | **85.8** | **92.0** | **94.3** | **94.5** | **94.5** | **94.5** | **99.6** | **94.7** | **98.6** | **99.7** |  | **99.8** | **99.8** | **99.4** | **99.5** | **96.4** | **74.7** | **74.6** | **74.8** | **Pi/SH/CH/050201/2012** |
| **23** | **84.4** | **86.3** | **87.5** | **85.4** | **85.8** | **87.3** | **83.9** | **87.6** | **85.3** | **85.6** | **83.0** | **85.7** | **91.9** | **94.2** | **94.5** | **94.5** | **94.5** | **99.6** | **94.6** | **98.5** | **99.7** | **99.9** |  | **100** | **99.3** | **99.4** | **96.4** | **74.7** | **74.6** | **74.7** | **Pi/SH/CH/051401/2012** |
| **24** | **84.4** | **86.3** | **87.5** | **85.4** | **85.8** | **87.3** | **83.9** | **87.6** | **85.3** | **85.6** | **83.0** | **85.7** | **91.9** | **94.2** | **94.5** | **94.5** | **94.5** | **99.6** | **94.6** | **98.5** | **99.7** | **99.9** | **100** |  | **99.3** | **99.4** | **96.4** | **74.7** | **74.6** | **74.7** | **Pi/SH/CH/051402/2012** |
| **25** | **84.5** | **86.4** | **87.6** | **85.5** | **85.9** | **87.4** | **83.9** | **87.7** | **85.4** | **85.8** | **83.0** | **85.8** | **92.1** | **94.5** | **94.7** | **94.7** | **94.7** | **99.9** | **94.9** | **98.8** | **99.9** | **99.7** | **99.7** | **99.7** |  | **99.7** | **96.9** | **74.9** | **74.8** | **74.9** | **Pi/SH/CH/061002/2011** |
| **26** | **84.5** | **86.4** | **87.6** | **85.5** | **85.8** | **87.4** | **84.0** | **87.6** | **85.4** | **85.7** | **83.0** | **85.8** | **92.0** | **94.4** | **94.6** | **94.6** | **94.6** | **99.8** | **94.8** | **98.7** | **99.8** | **99.7** | **99.7** | **99.7** | **99.8** |  | **96.7** | **74.9** | **74.8** | **74.8** | **Pi/SH/CH/120203/2011** |
| **27** | **84.1** | **85.9** | **87.2** | **85.1** | **85.4** | **86.9** | **83.7** | **87.2** | **85.0** | **85.4** | **82.7** | **85.4** | **91.3** | **93.6** | **94.0** | **94.0** | **94.0** | **98.5** | **94.1** | **97.8** | **98.6** | **98.3** | **98.3** | **98.3** | **98.5** | **98.5** |  | **74.0** | **73.9** | **74.0** | **pigeon/Ningxia/2068/2016** |
| **28** | **86.2** | **88.4** | **87.8** | **87.3** | **87.9** | **89.6** | **85.4** | **89.9** | **87.2** | **87.8** | **84.4** | **87.8** | **87.2** | **87.5** | **87.7** | **87.7** | **87.7** | **87.7** | **87.8** | **87.4** | **87.7** | **87.6** | **87.5** | **87.5** | **87.7** | **87.7** | **87.2** |  | **99.6** | **75.8** | **QH1** |
| **29** | **86.1** | **88.3** | **87.8** | **87.3** | **87.8** | **89.6** | **85.4** | **89.9** | **87.2** | **87.7** | **84.3** | **87.7** | **87.2** | **87.5** | **87.7** | **87.7** | **87.7** | **87.6** | **87.8** | **87.4** | **87.7** | **87.6** | **87.5** | **87.5** | **87.6** | **87.6** | **87.1** | **99.9** |  | **75.7** | **QH4** |
| **30** | **84.8** | **86.8** | **97.3** | **85.8** | **86.3** | **87.8** | **84.2** | **88.0** | **85.6** | **85.7** | **83.2** | **85.8** | **87.6** | **87.7** | **87.9** | **87.9** | **87.9** | **87.8** | **88.0** | **87.5** | **87.9** | **87.7** | **87.7** | **87.7** | **87.8** | **87.8** | **87.3** | **87.9** | **87.8** |  | **ZJ1** |

Note: Nucleic acid similarity was in the lower left corner；Amino acid similarity was in the upper right corner.

**Table S4**.The nucleotide sequence homology and amino acid homology comparisons of F gene of the eleven strains with reference strains (%)

|  | **1** | **2** | **3** | **4** | **5** | **6** | **7** | **8** | **9** | **10** | **11** | **12** | **13** | **14** | **15** | **16** | **17** | **18** | **19** | **20** | **21** | **22** | **23** | **24** | **25** | **26** | **27** | **28** | **29** | **30** | **31** | **32** | **33** | **34** | **35** | **36** | **37** | **38** | **39** | **40** | **41** | **42** | **43** | **44** |  |
| --- | --- | --- | --- | --- | --- | --- | --- | --- | --- | --- | --- | --- | --- | --- | --- | --- | --- | --- | --- | --- | --- | --- | --- | --- | --- | --- | --- | --- | --- | --- | --- | --- | --- | --- | --- | --- | --- | --- | --- | --- | --- | --- | --- | --- | --- |
| **1** |  | **90.4** | **90.8** | **90.6** | **90.8** | **89.9** | **95.0** | **90.8** | **92.4** | **95.0** | **96.9** | **94.8** | **94.8** | **94.4** | **92.2** | **96.2** | **94.2** | **86.8** | **90.6** | **91.3** | **91.3** | **91.5** | **91.2** | **91.2** | **91.0** | **90.6** | **91.2** | **91.5** | **90.6** | **90.6** | **90.6** | **90.6** | **90.6** | **90.4** | **90.8** | **91.7** | **90.8** | **90.1** | **91.0** | **92.4** | **92.1** | **92.4** | **91.0** | **90.5** | **98-1252** |
| **2** | **85.7** |  | **91.2** | **91.0** | **91.2** | **84.6** | **90.3** | **93.0** | **92.1** | **92.1** | **89.4** | **92.1** | **90.1** | **90.4** | **88.4** | **90.4** | **90.3** | **81.7** | **89.9** | **91.7** | **91.9** | **91.5** | **91.5** | **91.5** | **91.3** | **91.0** | **91.5** | **91.5** | **91.0** | **91.0** | **91.0** | **91.0** | **91.0** | **91.0** | **90.3** | **91.5** | **90.3** | **90.6** | **91.0** | **92.1** | **91.7** | **91.2** | **91.2** | **90.3** | **anhinga/U.S.(Fl)/44083/93** |
| **3** | **86.7** | **87.1** |  | **99.8** | **94.0** | **84.4** | **91.3** | **91.0** | **95.1** | **93.0** | **90.1** | **92.8** | **91.2** | **90.4** | **88.4** | **91.2** | **90.6** | **81.7** | **93.9** | **95.3** | **95.3** | **95.7** | **96.0** | **96.0** | **95.8** | **95.7** | **96.0** | **96.2** | **95.7** | **95.7** | **95.7** | **95.7** | **95.7** | **95.7** | **94.1** | **96.2** | **95.3** | **95.0** | **94.8** | **93.7** | **93.3** | **94.4** | **95.3** | **93.5** | **APMV1/Pigeon/NJ/USA/0721/2007** |
| **4** | **86.5** | **86.8** | **99.6** |  | **93.9** | **84.3** | **91.2** | **91.0** | **95.0** | **92.8** | **89.9** | **92.6** | **91.0** | **90.3** | **88.3** | **91.0** | **90.4** | **81.6** | **93.7** | **95.1** | **95.1** | **95.5** | **95.8** | **95.8** | **95.7** | **95.5** | **95.8** | **96.0** | **95.5** | **95.5** | **95.5** | **95.5** | **95.5** | **95.5** | **93.9** | **96.0** | **95.1** | **94.8** | **94.6** | **93.5** | **93.2** | **94.2** | **95.1** | **93.3** | **APMV1/Pigeon/PA/USA/0810/2008** |
| **5** | **86.7** | **87.5** | **89.2** | **89.2** |  | **85.4** | **91.9** | **91.0** | **94.6** | **93.5** | **90.5** | **93.0** | **91.7** | **91.2** | **88.6** | **91.4** | **91.0** | **83.0** | **92.8** | **94.0** | **94.0** | **95.0** | **94.9** | **94.9** | **94.8** | **93.9** | **94.9** | **94.6** | **93.9** | **93.9** | **93.9** | **93.9** | **93.9** | **93.7** | **93.1** | **94.8** | **93.3** | **93.1** | **93.9** | **93.3** | **93.0** | **93.9** | **94.6** | **97.3** | **chicken/China/Guangxi9/2003** |
| **6** | **73.5** | **70.8** | **71.5** | **71.4** | **71.7** |  | **88.4** | **84.8** | **85.9** | **88.6** | **90.3** | **87.7** | **88.3** | **87.7** | **88.4** | **89.7** | **87.5** | **94.2** | **84.4** | **852** | **85.2** | **85.9** | **85.9** | **85.9** | **85.7** | **85.7** | **85.9** | **85.5** | **85.7** | **85.7** | **85.7** | **85.7** | **85.7** | **85.7** | **84.4** | **85.7** | **84.** | **84.8** | **84.4** | **86.3** | **85.9** | **85.5** | **86.5** | **84.5** | **DE-R49/99** |
| **7** | **91.3** | **86.8** | **86.5** | **86.5** | **85.8** | **71.8** |  | **90.3** | **92.1** | **95.0** | **93.7** | **95.1** | **99.8** | **94.4** | **92.2** | **94.8** | **94.2** | **85.7** | **90.8** | **91.9** | **91.9** | **92.4** | **92.6** | **92.6** | **92.4** | **91.5** | **92.6** | **92.4** | **91.5** | **91.5** | **91.5** | **91.5** | **91.5** | **91.3** | **90.6** | **92.8** | **91.0** | **91.0** | **91.0** | **92.8** | **92.4** | **91.9** | **92.6** | **91.0** | **F48E8** |
| **8** | **85.8** | **91.3** | **86.2** | **86.2** | **86.0** | **72.3** | **86.0** |  | **92.6** | **91.3** | **89.7** | **91.5** | **90.1** | **89.4** | **88.1** | **90.1** | **89.5** | **82.3** | **89.7** | **915** | **91.5** | **90.8** | **91.3** | **91.3** | **91.2** | **90.8** | **91.3** | **91.5** | **90.8** | **90.8** | **90.8** | **90.8** | **90.8** | **90.8** | **90.1** | **91.9** | **90.5** | **90.5** | **90.5** | **92.4** | **92.1** | **91.9** | **91.2** | **90.3** | **gamefowl/U.S.(CA)/211472/02** |
| **9** | **87.1** | **88.2** | **93.4** | **93.3** | **90.7** | **72.5** | **87.3** | **87.7** |  | **93.5** | **91.5** | **93.3** | **91.9** | **91.5** | **89.5** | **92.2** | **91.3** | **82.8** | **94.6** | **95.9** | **95.9** | **95.8** | **95.5** | **95.5** | **95.3** | **95.9** | **95.5** | **96.2** | **95.8** | **95.8** | **95.8** | **95.8** | **95.8** | **95.7** | **94.8** | **96.4** | **95.3** | **95.1** | **96.0** | **94.2** | **93.9** | **98.2** | **95.3** | **94.0** | **GB 1168/84** |
| **10** | **91.8** | **88.4** | **89.2** | **89.0** | **89.1** | **72.5** | **93.2** | **87.7** | **89.6** |  | **93.3** | **98.6** | **94.8** | **95.3** | **91.9** | **95.3** | **95.1** | **85.4** | **92.6** | **92.6** | **92.6** | **93.5** | **93.5** | **93.5** | **93.3** | **92.8** | **93.5** | **93.3** | **92.8** | **92.8** | **92.8** | **92.8** | **92.8** | **92.6** | **92.8** | **93.0** | **91.9** | **92.2** | **92.6** | **94.0** | **93.7** | **93.1** | **93.5** | **92.6** | **Herts/33** |
| **11** | **95.2** | **84.1** | **86.3** | **86.3** | **86.0** | **73.5** | **90.1** | **84.6** | **86.4** | **90.3** |  | **93.2** | **93.5** | **92.4** | **92.4** | **96.6** | **92.6** | **87.2** | **89.7** | **90.8** | **90.4** | **90.6** | **90.3** | **90.3** | **90.1** | **89.9** | **90.3** | **90.8** | **89.9** | **89.9** | **89.9** | **89.9** | **89.9** | **89.7** | **89.9** | **90.8** | **89.5** | **89.4** | **90.1** | **92.1** | **91.7** | **91.3** | **90.3** | **89.5** | **I-2** |
| **12** | **91.8** | **88.6** | **89.5** | **89.4** | **89.2** | **72.5** | **93.1** | **87.8** | **89.8** | **98.7** | **90.3** |  | **95.0** | **94.8** | **91.9** | **95.3** | **94.6** | **84.5** | **91.7** | **92.4** | **92.4** | **93.3** | **93.3** | **93.3** | **93.1** | **92.2** | **93.3** | **93.1** | **92.2** | **92.2** | **92.2** | **92.2** | **92.2** | **92.1** | **91.9** | **92.8** | **91.7** | **92.1** | **92.1** | **93.7** | **93.3** | **93.0** | **93.0** | **92.1** | **Italien** |
| **13** | **91.3** | **86.7** | **86.5** | **86.5** | **86.8** | **71.9** | **99.8** | **85.9** | **87.3** | **93.1** | **90.1** | **93.0** |  | **94.2** | **92.1** | **94.6** | **94.1** | **85.6** | **90.6** | **91.7** | **91.7** | **92.3** | **92.4** | **92.4** | **92.2** | **91.3** | **92.4** | **92.2** | **91.3** | **91.3** | **91.3** | **91.3** | **91.3** | **91.2** | **90.4** | **92.6** | **90.8** | **90.8** | **90.8** | **92.6** | **92.2** | **91.7** | **92.4** | **90.8** | **JS/1/02/Du** |
| **14** | **90.9** | **86.2** | **86.2** | **86.0** | **86.8** | **72.9** | **92.1** | **85.6** | **86.7** | **93.1** | **89.7** | **92.9** | **92.1** |  | **90.1** | **94.4** | **99.8** | **85.2** | **90.3** | **90.6** | **90.6** | **91.9** | **91.7** | **91.7** | **91.5** | **90.3** | **91.7** | **91.0** | **90.3** | **90.3** | **90.3** | **90.3** | **90.3** | **90.1** | **89.9** | **91.5** | **89.9** | **89.7** | **90.1** | **92.2** | **91.9** | **91.3** | **91.2** | **90.3** | **JS/9/05/Go** |
| **15** | **88.9** | **85.1** | **85.1** | **85.0** | **84.3** | **71.9** | **89.3** | **83.9** | **85.3** | **89.3** | **88.3** | **89.3** | **89.2** | **88.5** |  | **93.9** | **90.3** | **85.4** | **88.3** | **89.2** | **89.2** | **89.0** | **89.5** | **89.5** | **89.4** | **89.0** | **89.5** | **89.7** | **89.0** | **89.0** | **89.0** | **89.0** | **89.0** | **88.8** | **88.6** | **89.5** | **88.6** | **88.4** | **88.3** | **90.3** | **89.9** | **89.7** | **89.4** | **87.9** | **LaSota** |
| **16** | **90.6** | **85.2** | **85.8** | **85.7** | **85.4** | **73.3** | **90.0** | **84.3** | **85.9** | **90.4** | **89.8** | **90.4** | **89.9** | **89.4** | **90.8** |  | **94.6** | **87.0** | **90.4** | **91.3** | **91.3** | **92.1** | **92.1** | **92.1** | **91.9** | **91.2** | **92.1** | **92.1** | **91.2** | **91.2** | **91.2** | **91.2** | **91.2** | **91.0** | **90.4** | **91.3** | **90.3** | **90.6** | **90.8** | **92.8** | **92.4** | **92.1** | **91.9** | **90.5** | **mallard/US(OH)/86-233/1986** |
| **17** | **90.9** | **86.2** | **86.3** | **86.2** | **86.8** | **72.8** | **92.1** | **85.7** | **86.7** | **93.1** | **89.7** | **92.9** | **92.1** | **99.9** | **88.6** | **89.5** |  | **85.0** | **90.1** | **90.4** | **90.4** | **91.7** | **91.5** | **91.5** | **91.3** | **90.3** | **91.5** | **91.0** | **90.3** | **90.3** | **90.3** | **90.3** | **90.3** | **90.1** | **89.7** | **91.3** | **89.7** | **89.7** | **89.9** | **92.4** | **92.1** | **91.2** | **91.0** | **90.1** | **Mukteswar** |
| **18** | **72.4** | **69.5** | **70.8** | **70.5** | **71.0** | **93.0** | **70.8** | **70.8** | **71.7** | **71.1** | **72.2** | **71.4** | **70.8** | **71.8** | **70.7** | **72.2** | **71.7** |  | **82.1** | **82.1** | **82.1** | **83.0** | **83.0** | **83.0** | **82.8** | **82.8** | **83.0** | **82.6** | **82.8** | **82.8** | **828** | **82.8** | **82.8** | **82.8** | **81.7** | **83.2** | **81.7** | **82.1** | **81.6** | **84.1** | **83.7** | **82.6** | **83.4** | **82.1** | **NDV08-004** |
| **19** | **86.3** | **86.8** | **92.3** | **92.2** | **88.9** | **71.3** | **86.5** | **86.6** | **94.0** | **88.8** | **85.4** | **88.6** | **86.6** | **85.5** | **84.2** | **85.4** | **85.5** | **70.7** |  | **93.3** | **93.3** | **93.9** | **94.0** | **94.0** | **93.9** | **94.1** | **94.0** | **94.6** | **94.0** | **94.0** | **94.0** | **94.0** | **94.0** | **93.9** | **98.2** | **94.2** | **93.2** | **93.3** | **95.3** | **93.0** | **92.6** | **94.1** | **93.7** | **92.4** | **PG/CH/JS/1/05** |
| **20** | **85.7** | **87.2** | **93.5** | **93.2** | **88.6** | **72.4** | **86.0** | **86.3** | **93.5** | **88.0** | **85.1** | **88.2** | **86.0** | **85.9** | **84.6** | **84.8** | **85.8** | **71.3** | **92.1** |  | **99.6** | **96.0** | **96.0** | **96.0** | **95.8** | **96.4** | **96.0** | **96.6** | **96.4** | **96.4** | **96.4** | **96.4** | **96.4** | **96.2** | **93.5** | **97.1** | **95.3** | **95.9** | **95.1** | **93.0** | **92.6** | **95.3** | **96.0** | **93.5** | **PHL264752** |
| **21** | **85.7** | **87.1** | **93.7** | **93.7** | **88.9** | **72.2** | **86.1** | **86.4** | **94.1** | **88.0** | **84.8** | **88.2** | **86.2** | **85.7** | **84.0** | **84.8** | **85.7** | **71.0** | **92.7** | **98.8** |  | **96.0** | **96.0** | **96.0** | **95.8** | **96.4** | **96.0** | **96.6** | **96.4** | **96.4** | **96.4** | **96.4** | **96.4** | **96.2** | **93.5** | **97.1** | **95.3** | **95.9** | **95.0** | **93.0** | **92.6** | **95.3** | **96.0** | **93.5** | **PHL37114.2** |
| **22** | **85.7** | **86.8** | **94.6** | **94.5** | **89.8** | **72.0** | **86.5** | **86.1** | **94.0** | **88.4** | **85.2** | **88.6** | **86.5** | **86.1** | **84.6** | **85.6** | **86.1** | **71.2** | **92.8** | **93.3** | **93.8** |  | **97.8** | **97.8** | **97.7** | **96.9** | **97.8** | **97.1** | **96.9** | **96.9** | **96.9** | **96.9** | **96.9** | **96.8** | **93.7** | **96.6** | **95.1** | **96.2** | **95.3** | **93.1** | **93.0** | **95.1** | **98.6** | **93.9** | **Pi/CH/LHLJ/110813** |
| **23** | **86.0** | **86.8** | **95.1** | **95.0** | **89.6** | **72.0** | **86.9** | **86.4** | **93.8** | **88.5** | **85.4** | **88.9** | **86.9** | **86.2** | **84.7** | **86.0** | **86.2** | **71.3** | **92.7** | **93.2** | **93.7** | **97.0** |  | **100** | **99.8** | **96.9** | **100** | **97.5** | **96.9** | **96.9** | **96.9** | **96.9** | **96.9** | **96.8** | **94.0** | **96.6** | **94.6** | **96.2** | **95.3** | **93.3** | **93.1** | **94.8** | **97.5** | **93.9** | **Pi/SH/CH/010502/2010** |
| **24** | **86.0** | **86.8** | **95.1** | **95.0** | **89.6** | **72.0** | **86.9** | **86.4** | **93.8** | **88.5** | **85.4** | **88.9** | **86.9** | **86.2** | **84.7** | **86.0** | **86.2** | **71.3** | **92.7** | **93.2** | **93.7** | **97.0** | **100** |  | **99.8** | **96.9** | **100** | **97.5** | **96.9** | **96.9** | **96.9** | **96.9** | **96.9** | **96.8** | **94.0** | **96.6** | **94.6** | **96.2** | **95.3** | **93.3** | **93.1** | **94.8** | **97.5** | **93.9** | **Pi/SH/CH/010515/2010** |
| **25** | **86.0** | **86.7** | **95.1** | **94.9** | **89.6** | **72.0** | **86.9** | **86.3** | **93.7** | **88.4** | **85.4** | **88.8** | **86.9** | **86.1** | **84.7** | **86.0** | **86.1** | **71.3** | **92.6** | **93.1** | **93.6** | **97.0** | **99.9** | **99.9** |  | **96.8** | **99.8** | **97.3** | **96.8** | **96.8** | **96.8** | **96.8** | **96.8** | **96.6** | **93.9** | **96.4** | **94.4** | **96.0** | **95.5** | **93.1** | **93.0** | **94.6** | **97.3** | **93.7** | **Pi/SH/CH/010516/2010** |
| **26** | **85.3** | **86.3** | **94.1** | **94.1** | **88.3** | **72.0** | **85.9** | **85.9** | **93.6** | **88.0** | **84.6** | **88.1** | **86.0** | **85.5** | **84.1** | **85.2** | **85.5** | **71.0** | **92.2** | **93.1** | **94.0** | **95.5** | **95.7** | **95.7** | **95.6** |  | **96.9** | **98.7** | **100** | **100** | **100** | **100** | **100** | **99.8** | **94.1** | **96.6** | **94.6** | **98.9** | **95.7** | **93.0** | **92.6** | **95.1** | **97.7** | **93.1** | **Pi/SH/CH/0167/2013** |
| **27** | **86.1** | **86.8** | **95.2** | **95.1** | **89.5** | **72.0** | **87.0** | **86.5** | **93.9** | **88.6** | **85.5** | **89.0** | **87.0** | **86.3** | **84.9** | **86.0** | **86.3** | **71.3** | **92.8** | **93.3** | **93.8** | **97.2** | **99.9** | **99.9** | **99.8** | **95.8** |  | **97.5** | **96.9** | **96.9** | **96.9** | **96.9** | **96.9** | **96.8** | **94.0** | **96.6** | **94.6** | **96.2** | **95.3** | **93.3** | **93.1** | **94.8** | **97.5** | **93.9** | **Pi/SH/CH/022401/2009** |
| **28** | **85.4** | **86.0** | **93.8** | **93.7** | **88.2** | **71.7** | **85.7** | **85.7** | **93.2** | **87.7** | **84.4** | **87.9** | **85.8** | **85.5** | **83.8** | **84.5** | **85.5** | **71.0** | **91.8** | **92.7** | **93.5** | **95.1** | **95.3** | **95.3** | **95.2** | **98.4** | **95.4** |  | **98.7** | **98.7** | **98.7** | **98.7** | **98.7** | **98.6** | **94.8** | **97.1** | **95.3** | **98.0** | **96.0** | **93.5** | **93.1** | **95.8** | **97.5** | **94.2** | **Pi/SH/CH/040601/2012** |
| **29** | **85.4** | **86.4** | **94.2** | **94.2** | **88.4** | **72.0** | **86.0** | **86.0** | **93.6** | **88.0** | **84.7** | **88.1** | **86.1** | **85.5** | **84.2** | **85.1** | **85.6** | **71.0** | **92.3** | **93.1** | **94.0** | **95.5** | **95.7** | **95.7** | **95.7** | **99.9** | **95.8** | **98.5** |  | **100** | **100** | **100** | **100** | **99.8** | **94.0** | **96.6** | **94.6** | **98.9** | **95.7** | **93.0** | **92.6** | **95.1** | **97.7** | **93.1** | **Pi/SH/CH/041002/2011** |
| **30** | **85.4** | **86.4** | **94.1** | **94.1** | **88.4** | **71.9** | **86.0** | **86.0** | **93.6** | **88.0** | **84.7** | **88.1** | **86.1** | **85.5** | **84.2** | **85.1** | **85.6** | **71.0** | **92.2** | **93.1** | **93.9** | **95.5** | **95.7** | **95.7** | **95.6** | **99.9** | **95.8** | **98.4** | **99.9** |  | **100** | **100** | **100** | **99.8** | **94.0** | **96.6** | **94.6** | **98.9** | **95.7** | **93.0** | **92.6** | **95.1** | **97.7** | **93.1** | **Pi/SH/CH/050201/2012** |
| **31** | **85.4** | **86.4** | **94.1** | **94.1** | **88.4** | **71.9** | **86.0** | **86.0** | **93.6** | **88.0** | **84.7** | **88.1** | **86.1** | **85.5** | **84.2** | **85.1** | **85.6** | **71.0** | **92.2** | **93.1** | **93.9** | **95.5** | **95.7** | **95.7** | **95.6** | **99.9** | **95.8** | **98.4** | **99.9** | **100** |  | **100** | **100** | **99.8** | **94.0** | **96.6** | **94.6** | **98.9** | **95.7** | **93.0** | **92.6** | **95.1** | **97.7** | **93.1** | **Pi/SH/CH/051401/2012** |
| **32** | **85.4** | **86.4** | **94.1** | **94.1** | **88.4** | **71.9** | **86.0** | **86.0** | **93.6** | **88.0** | **84.7** | **88.1** | **86.1** | **85.5** | **84.2** | **85.1** | **85.6** | **71.0** | **92.2** | **93.1** | **93.9** | **95.5** | **95.7** | **95.7** | **95.6** | **99.9** | **95.8** | **98.4** | **99.9** | **100** | **100** |  | **100** | **99.8** | **94.0** | **96.6** | **94.6** | **98.9** | **95.7** | **93.0** | **92.6** | **95.1** | **97.7** | **93.1** | **Pi/SH/CH/051402/2012** |
| **33** | **85.4** | **86.4** | **94.2** | **94.2** | **88.4** | **72.0** | **86.0** | **86.0** | **93.6** | **88.0** | **84.7** | **88.1** | **86.1** | **85.5** | **84.2** | **85.1** | **85.6** | **71.0** | **92.3** | **93.1** | **94.0** | **95.5** | **95.7** | **95.7** | **95.7** | **99.9** | **95.8** | **98.5** | **100** | **99.9** | **99.9** | **99.9** |  | **99.8** | **94.0** | **96.6** | **94.6** | **98.9** | **95.7** | **93.0** | **92.6** | **95.1** | **97.7** | **93.1** | **Pi/SH/CH/061002/2011** |
| **34** | **85.3** | **86.3** | **94.2** | **94.2** | **88.3** | **72.0** | **85.9** | **85.9** | **93.6** | **88.0** | **84.6** | **88.1** | **85.0** | **85.5** | **84.1** | **85.1** | **85.5** | **71.0** | **92.2** | **93.1** | **93.9** | **95.5** | **95.7** | **95.7** | **95.6** | **99.9** | **95.8** | **98.4** | **99.9** | **99.9** | **99.9** | **99.9** | **99.9** |  | **93.9** | **96.4** | **94.4** | **98.7** | **95.5** | **93.0** | **92.6** | **94.9** | **97.5** | **93.0** | **Pi/SH/CH/120203/2011** |
| **35** | **86.2** | **87.1** | **92.7** | **92.6** | **89.2** | **71.3** | **86.4** | **87.0** | **94.2** | **88.7** | **85.4** | **88.6** | **86.5** | **85.5** | **84.0** | **85.3** | **85.5** | **70.5** | **98.4** | **92.3** | **92.9** | **92.9** | **93.0** | **93.0** | **93.0** | **92.5** | **93.1** | **92.2** | **92.5** | **92.5** | **92.5** | **92.5** | **92.5** | **92.5** |  | **94.2** | **93.3** | **93.5** | **95.7** | **92.6** | **92.3** | **94.6** | **93.7** | **93.1** | **Pigeon/China/100/08** |
| **36** | **85.3** | **86.8** | **93.7** | **93.7** | **88.7** | **72.0** | **86.4** | **86.7** | **93.6** | **87.6** | **84.5** | **88.0** | **86.4** | **85.6** | **83.9** | **84.5** | **85.6** | **71.4** | **92.2** | **93.7** | **94.2** | **93.7** | **93.9** | **93.9** | **93.8** | **93.0** | **93.9** | **92.8** | **93.1** | **93.0** | **93.0** | **93.0** | **93.1** | **93.0** | **92.5** |  | **96.8** | **95.9** | **95.9** | **93.9** | **93.5** | **96.2** | **96.2** | **94.0** | **pigeon/Nigeria/NIE09-1898/2009** |
| **37** | **85.4** | **86.5** | **92.9** | **93.0** | **88.8** | **71.6** | **86.1** | **86.1** | **92.9** | **87.4** | **84.6** | **87.6** | **86.1** | **84.8** | **84.3** | **84.6** | **84.8** | **71.0** | **91.3** | **92.2** | **92.5** | **92.8** | **92.2** | **92.2** | **92.2** | **91.9** | **92.2** | **91.5** | **91.9** | **91.9** | **91.9** | **91.9** | **91.9** | **91.9** | **91.5** | **94.0** |  | **93.9** | **94.1** | **93.5** | **93.2** | **95.0** | **94.2** | **92.8** | **pigeon/Nigeria/NIE13-092/2013** |
| **38** | **84.9** | **86.3** | **93.5** | **93.4** | **88.0** | **717** | **85.2** | **85.4** | **93.0** | **87.3** | **84.2** | **87.5** | **85.4** | **84.8** | **83.7** | **84.6** | **84.9** | **70.6** | **91.4** | **92.3** | **93.1** | **94.6** | **94.8** | **94.8** | **94.8** | **98.8** | **94.9** | **97.5** | **98.9** | **98.8** | **98.8** | **98.8** | **98.9** | **98.8** | **91.7** | **92.3** | **91.1** |  | **95.3** | **92.4** | **92.1** | **94.4** | **96.9** | **92.8** | **pigeon/Ningxia/2068/2016** |
| **39** | **86.0** | **87.2** | **92.8** | **92.7** | **89.7** | **70.8** | **86.0** | **86.6** | **94.4** | **88.3** | **85.3** | **88.4** | **86.0** | **85.8** | **84.0** | **84.4** | **85.8** | **69.9** | **93.7** | **92.8** | **93.0** | **93.1** | **92.9** | **92.9** | **93.0** | **92.8** | **93.0** | **92.3** | **92.8** | **92.8** | **92.8** | **92.8** | **92.8** | **92.8** | **94.0** | **92.8** | **91.7** | **92.0** |  | **93.0** | **92.8** | **95.3** | **95.7** | **93.1** | **Pigeon/Texas/209682/2002** |
| **40** | **88.2** | **88.6** | **89.8** | **89.5** | **88.9** | **72.2** | **88.6** | **88.0** | **90.6** | **90.8** | **86.9** | **90.6** | **88.5** | **88.2** | **86.1** | **87.1** | **88.3** | **72.1** | **89.2** | **89.5** | **89.5** | **89.4** | **89.3** | **89.3** | **89.3** | **89.2** | **89.5** | **89.1** | **89.3** | **89.3** | **89.3** | **89.3** | **89.3** | **89.3** | **89.1** | **89.2** | **88.2** | **88.8** | **88.9** |  | **99.6** | **93.7** | **93.0** | **92.8** | **QH1** |
| **41** | **88.1** | **88.5** | **89.7** | **89.4** | **88.8** | **72.1** | **88.5** | **87.9** | **90.5** | **90.7** | **86.8** | **90.4** | **88.4** | **88.1** | **86.0** | **87.0** | **88.2** | **72.0** | **89.1** | **89.4** | **89.4** | **89.3** | **89.2** | **89.2** | **89.2** | **89.1** | **89.3** | **89.0** | **89.2** | **89.2** | **89.2** | **89.2** | **89.2** | **89.2** | **88.9** | **89.1** | **88.1** | **88.7** | **88.8** | **99.9** |  | **93.3** | **92.8** | **92.4** | **QH4** |
| **42** | **87.0** | **87.7** | **93.2** | **93.1** | **90.1** | **72.8** | **87.1** | **87.7** | **98.1** | **89.5** | **86.2** | **89.7** | **87.0** | **86.8** | **85.2** | **86.0** | **86.8** | **71.8** | **93.7** | **93.5** | **93.9** | **93.9** | **93.6** | **93.6** | **93.5** | **93.4** | **93.7** | **93.1** | **93.5** | **93.4** | **93.4** | **93.4** | **93.5** | **93.4** | **94.1** | **93.5** | **92.6** | **92.6** | **94.2** | **90.5** | **90.4** |  | **94.6** | **93.7** | **s-1** |
| **43** | **85.4** | **86.6** | **94.3** | **94.3** | **88.9** | **71.9** | **86.3** | **86.0** | **93.1** | **88.2** | **85.0** | **88.3** | **86.4** | **85.7** | **84.6** | **85.3** | **85.7** | **70.8** | **92.3** | **93.1** | **93.4** | **97.9** | **96.6** | **96.6** | **96.6** | **95.6** | **96.7** | **95.0** | **95.7** | **95.6** | **95.6** | **95.6** | **95.7** | **95.6** | **92.4** | **93.3** | **92.2** | **94.8** | **92.7** | **89.1** | **89.0** | **93.1** |  | **93.5** | **sms12** |
| **44** | **86.2** | **87.3** | **89.3** | **89.2** | **97.8** | **71.1** | **86.8** | **85.7** | **90.9** | **88.7** | **85.5** | **88.7** | **86.7** | **86.4** | **84.3** | **85.2** | **86.4** | **70.5** | **89.1** | **88.5** | **89.0** | **89.5** | **89.7** | **89.7** | **89.6** | **88.5** | **89.7** | **88.4** | **88.6** | **88.6** | **88.6** | **88.6** | **88.6** | **88.5** | **89.5** | **88.6** | **88.9** | **88.3** | **89.1** | **88.7** | **88.6** | **90.3** | **88.8** |  | **ZJ1** |

Note: Nucleic acid similarity was in the lower left corner；Amino acid similarity was in the upper right corner.

**Table S5**.The nucleotide sequence homology and amino acid homology comparisons of HN gene of the eleven strains with reference strains (%)

|  | **1** | **2** | **3** | **4** | **5** | **6** | **7** | **8** | **9** | **10** | **11** | **12** | **13** | **14** | **15** | **16** | **17** | **18** | **19** | **20** | **21** | **22** | **23** | **24** | **25** | **26** | **27** | **28** | **29** | **30** | **31** | **32** | **33** | **34** |  |
| --- | --- | --- | --- | --- | --- | --- | --- | --- | --- | --- | --- | --- | --- | --- | --- | --- | --- | --- | --- | --- | --- | --- | --- | --- | --- | --- | --- | --- | --- | --- | --- | --- | --- | --- | --- |
| **1** |  | **88.7** | **90.6** | **85.2** | **92.8** | **90.1** | **92.7** | **95.3** | **93.2** | **92.8** | **92.0** | **94.1** | **96.7** | **92.5** | **83.3** | **89.2** | **89.5** | **88.0** | **88.6** | **88.6** | **88.6** | **89.2** | **88.8** | **89.2** | **89.2** | **89.2** | **89.0** | **89.0** | **89.2** | **89.2** | **89.3** | **90.8** | **90.2** | **89.9** | **98-1252** |
| **2** | **84.5** |  | **91.3** | **83.2** | **89.0** | **95.5** | **90.1** | **88.0** | **90.8** | **88.8** | **88.8** | **88.5** | **89.2** | **88.8** | **82.0** | **90.1** | **90.8** | **90.2** | **90.9** | **90.9** | **90.9** | **90.2** | **91.1** | **89.7** | **90.2** | **90.4** | **90.2** | **90.2** | **90.2** | **90.2** | **89.9** | **93.9** | **93.4** | **92.0** | **anhinga/U.S.(Fl)/44083/93** |
| **3** | **86.8** | **87.0** |  | **83.8** | **90.4** | **92.3** | **91.4** | **89.2** | **92.0** | **90.2** | **90.2** | **89.2** | **91.1** | **90.8** | **82.7** | **91.4** | **91.8** | **90.2** | **90.9** | **90.9** | **90.9** | **91.1** | **91.1** | **91.1** | **91.1** | **91.1** | **90.9** | **90.9** | **91.1** | **91.1** | **90.9** | **93.4** | **92.8** | **96.5** | **chicken/China/Guangxi9/2003** |
| **4** | **71.7** | **72.4** | **72.1** |  | **85.9** | **83.4** | **85.2** | **84.7** | **84.8** | **86.0** | **85.0** | **84.6** | **85.9** | **85.2** | **94.2** | **83.1** | **83.8** | **82.9** | **83.2** | **83.2** | **83.2** | **83.6** | **83.4** | **83.6** | **83.6** | **83.6** | **83.4** | **83.4** | **83.6** | **83.6** | **83.6** | **84.8** | **84.3** | **84.3** | **DE-R49/99** |
| **5** | **90.0** | **85.9** | **86.6** | **72.3** |  | **90.1** | **93.2** | **92.1** | **94.1** | **99.3** | **92.1** | **91.3** | **93.7** | **92.8** | **84.** | **89.4** | **90.1** | **88.5** | **89.3** | **89.3** | **89.3** | **89.2** | **89.5** | **89.2** | **89.2** | **89.2** | **89.0** | **89.0** | **89.2** | **89.2** | **89.2** | **91.1** | **90.6** | **89.5** | **F48E8** |
| **6** | **85.8** | **91.8** | **88.0** | **71.0** | **86.9** |  | **90.9** | **89.2** | **91.3** | **89.9** | **89.5** | **89.5** | **90.6** | **89.5** | **82.9** | **90.4** | **90.8** | **90.8** | **91.4** | **91.4** | **91.4** | **90.6** | **91.6** | **90.2** | **90.6** | **90.6** | **90.4** | **90.4** | **90.6** | **90.6** | **90.4** | **93.5** | **93.0** | **92.3** | **gamefowl/U.S.(CA)/211472/02** |
| **7** | **89.8** | **88.2** | **88.5** | **71.9** | **91.0** | **88.7** |  | **91.8** | **98.1** | **93.2** | **92.8** | **91.8** | **937** | **93.2** | **82.9** | **90.2** | **90.9** | **89.0** | **89.9** | **89.9** | **89.9** | **89.7** | **90.0** | **89.2** | **89.7** | **897** | **89.5** | **895** | **897** | **897** | **89.5** | **92.7** | **92.1** | **91.1** | **Herts/33** |
| **8** | **94.7** | **84.2** | **85.9** | **72.4** | **88.5** | **85.0** | **88.5** |  | **92.1** | **92** | **91.4** | **93.4** | **955** | **91.8** | **83.1** | **88.0** | **88.7** | **87.4** | **87.8** | **87.8** | **87.8** | **88.3** | **87.9** | **885** | **883** | **88.3** | **88.1** | **88.1** | **88.3** | **88.3** | **885** | **90.4** | **89.9** | **88.5** | **I-2** |
| **9** | **90.4** | **88.4** | **89.0** | **72.1** | **91.4** | **88.9** | **98.1** | **89.1** |  | **94.1** | **93.4** | **91.6** | **93.9** | **93.9** | **82.9** | **90.4** | **91.1** | **89.5** | **90.4** | **90.4** | **90.4** | **90.2** | **90.6** | **89.7** | **90.2** | **90.2** | **90.0** | **90.0** | **90.2** | **90.2** | **90.0** | **93.0** | **92.5** | **91.1** | **Italien** |
| **10** | **90.1** | **85.9** | **86.8** | **72.5** | **99.6** | **86.9** | **91.0** | **88.6** | **91.5** |  | **92.1** | **91.3** | **93.7** | **92.8** | **84.5** | **89.2** | **89.9** | **88.3** | **89.2** | **89.2** | **89.2** | **89.0** | **89.3** | **89.0** | **89.0** | **89.0** | **88.8** | **88.8** | **89.0** | **89.0** | **89.0** | **90.9** | **90.4** | **89.4** | **JS/1/02/Du** |
| **11** | **90.6** | **85.9** | **89.3** | **72.6** | **91.4** | **86.6** | **92.1** | **89.4** | **92.5** | **91.6** |  | **90.4** | **93.2** | **99.0** | **83.1** | **88.3** | **89.0** | **88.3** | **89.2** | **89.2** | **89.2** | **88.5** | **89.3** | **88.5** | **88.5** | **88.5** | **88.3** | **88.3** | **88.5** | **88.5** | **88.6** | **91.1** | **90.8** | **89.2** | **JS/9/05/Go** |
| **12** | **89.5** | **83.0** | **84.2** | **71.9** | **88.3** | **83.8** | **87.9** | **88.5** | **88.2** | **88.4** | **88.3** |  | **94.8** | **90.4** | **83.4** | **88.5** | **88.6** | **87.4** | **88.1** | **88.1** | **88.1** | **88.6** | **88.3** | **88.6** | **88.6** | **88.6** | **88.5** | **88.5** | **88.6** | **88.6** | **88.3** | **90.4** | **90.2** | **88.8** | **LaSota** |
| **13** | **91.3** | **83.3** | **85.4** | **73.2** | **89.3** | **84.9** | **89.2** | **90.4** | **89.3** | **89.3** | **89.9** | **90.4** |  | **93.2** | **82.5** | **90.1** | **90.8** | **89.0** | **89.5** | **89.5** | **89.5** | **90.0** | **89.7** | **90.0** | **90.0** | **90.0** | **89.9** | **89.9** | **90.0** | **90.0** | **90.2** | **91.6** | **91.1** | **90.6** | **mallard/US(OH)/86-233/1986** |
| **14** | **90.8** | **86.1** | **89.4** | **72.6** | **91.6** | **86.7** | **92.2** | **89.4** | **92.8** | **91.8** | **99.7** | **88.2** | **89.9** |  | **83.2** | **88.7** | **89.4** | **88.7** | **89.5** | **89.5** | **89.5** | **88.8** | **89.7** | **88.8** | **88.8** | **88.8** | **88.6** | **88.6** | **88.8** | **88.8** | **89.0** | **91.3** | **90.8** | **89.4** | **Mukteswar** |
| **15** | **71.2** | **71.9** | **71.2** | **92.2** | **71.6** | **70.7** | **71.5** | **71.7** | **71.6** | **71.8** | **71.7** | **71.6** | **71.6** | **71.7** |  | **82.0** | **82.7** | **82.2** | **82.5** | **82.5** | **82.5** | **82.5** | **82.7** | **82.7** | **82.5** | **82.5** | **82.3** | **82.3** | **82.5** | **82.5** | **82.5** | **83.6** | **83.1** | **83.2** | **NDV08-004** |
| **16** | **85.2** | **86.1** | **87.8** | **71.8** | **85.2** | **87.5** | **87.7** | **84.7** | **88.4** | **85.1** | **85.8** | **82.7** | **84.0** | **85.9** | **70.8** |  | **993** | **94.6** | **95.3** | **95.3** | **95.3** | **94.8** | **95.5** | **94.2** | **94.8** | **94.4** | **94.6** | **94.6** | **94.8** | **94.8** | **94.2** | **91.6** | **91.1** | **92.1** | **PHL264752** |
| **17** | **85.9** | **86.6** | **88.3** | **71.7** | **85.7** | **87.8** | **88.4** | **85.2** | **89.1** | **85.6** | **86.3** | **82.9** | **84.6** | **86.4** | **70.8** | **98.9** |  | **95.3** | **96.0** | **96.0** | **96.0** | **955** | **96.2** | **94.9** | **95.5** | **95.1** | **95.3** | **95.3** | **95.5** | **95.5** | **94.9** | **92.3** | **91.8** | **92.5** | **PHL37114.2** |
| **18** | **83.8** | **85.6** | **86.6** | **72.1** | **84.1** | **86.0** | **86.3** | **83.9** | **87.0** | **84.0** | **85.2** | **82.1** | **83.3** | **85.3** | **71.2** | **91.7** | **92.3** |  | **97.9** | **97.9** | **97.9** | **95.8** | **98.1** | **95.3** | **95.8** | **95.5** | **95.6** | **95.6** | **95.8** | **95.8** | **95.3** | **92.0** | **91.4** | **90.6** | **Pi/CH/LHLJ/110813** |
| **19** | **84.6** | **86.7** | **875** | **71.9** | **85.4** | **87.1** | **87.4** | **84.4** | **88.0** | **85.3** | **86.3** | **82.9** | **83.5** | **86.4** | **71.3** | **92.4** | **93.0** | **96.3** |  | **100** | **100** | **97.4** | **99.8** | **96.9** | **97.4** | **97.0** | **97.2** | **97.2** | **97.4** | **97.4** | **96.9** | **92.8** | **92.3** | **91.3** | **Pi/SH/CH/010502/2010** |
| **20** | **84.6** | **86.7** | **87.5** | **71.9** | **85.4** | **87.1** | **87.4** | **84.4** | **88.0** | **85.3** | **86.3** | **82.9** | **83.5** | **86.4** | **71.3** | **92.4** | **93.0** | **96.3** | **100.0** |  | **100** | **97.4** | **99.8** | **96.9** | **97.4** | **97.0** | **97.2** | **97.2** | **97.4** | **97.4** | **96.9** | **92.8** | **92.3** | **91.3** | **Pi/SH/CH/010515/2010** |
| **21** | **84.6** | **86.7** | **87.5** | **71.9** | **85.4** | **87.1** | **87.4** | **84.4** | **88.0** | **85.3** | **86.3** | **82.9** | **83.5** | **86.4** | **71.3** | **92.4** | **93.0** | **96.3** | **100.0** | **100.0** |  | **97.4** | **99.8** | **96.9** | **97.4** | **97.0** | **97.2** | **97.2** | **97.4** | **97.4** | **96.9** | **92.8** | **92.3** | **91.3** | **Pi/SH/CH/010516/2010** |
| **22** | **85.1** | **86.5** | **87.7** | **72.0** | **85.3** | **86.8** | **86.8** | **84.6** | **87.6** | **85.2** | **85.6** | **83.6** | **83.7** | **85.7** | **71.6** | **92.0** | **92.6** | **93.8** | **95.1** | **95.1** | **95.1** |  | **97.6** | **99.5** | **100** | **99.7** | **99.8** | **99.8** | **100** | **100** | **99.1** | **92.1** | **91.6** | **91.6** | **Pi/SH/CH/0167/2013** |
| **23** | **84.6** | **86.8** | **87.5** | **72.0** | **85.5** | **87.2** | **87.4** | **84.5** | **88.1** | **85.3** | **86.3** | **82.9** | **83.6** | **86.4** | **71.4** | **92.4** | **93.1** | **96.4** | **99.9** | **99.9** | **99.9** | **95.2** |  | **97.0** | **97.6** | **97.2** | **97.4** | **97.4** | **97.6** | **97.6** | **97.0** | **93.0** | **92.5** | **91.4** | **Pi/SH/CH/022401/2009** |
| **24** | **84.8** | **85.8** | **87.7** | **72.1** | **85.2** | **86.3** | **86.5** | **84.5** | **87.3** | **85.0** | **85.5** | **83.5** | **83.6** | **85.5** | **71.6** | **91.8** | **92.5** | **93.6** | **94.9** | **94.9** | **94.9** | **99.2** | **95.0** |  | **99.5** | **99.1** | **99.3** | **99.3** | **99.5** | **99.5** | **98.6** | **91.6** | **91.1** | **91.4** | **Pi/SH/CH/040601/2012** |
| **25** | **85.2** | **86.5** | **87.7** | **72.0** | **85.3** | **86.8** | **86.9** | **84.7** | **87.7** | **85.2** | **85.6** | **83.6** | **83.7** | **85.7** | **71.6** | **92.1** | **92.7** | **93.8** | **95.2** | **95.2** | **95.2** | **99.9** | **95.2** | **99.3** |  | **99.7** | **99.8** | **99.8** | **100** | **100** | **99.1** | **92.1** | **91.6** | **91.6** | **Pi/SH/CH/041002/2011** |
| **26** | **85.0** | **86.3** | **87.6** | **72.0** | **85.2** | **86.6** | **86.7** | **84.5** | **87.5** | **85.0** | **85.5** | **83.5** | **83.5** | **85.6** | **71.6** | **91.9** | **92.5** | **93.5** | **94.9** | **94.9** | **94.9** | **99.8** | **94.9** | **99.0** | **99.7** |  | **99.8** | **99.8** | **99.7** | **99.7** | **98.8** | **92.1** | **91.6** | **91.6** | **Pi/SH/CH/050201/2012** |
| **27** | **84.8** | **86.3** | **87.4** | **72.0** | **85.0** | **86.5** | **86.5** | **84.3** | **87.3** | **84.9** | **85.3** | **83.5** | **83.4** | **85.4** | **71.7** | **91.7** | **92.4** | **93.5** | **94.8** | **94.8** | **94.8** | **997** | **94.9** | **98.9** | **99.6** | **997** |  | **100** | **99.8** | **99.8** | **99.0** | **92.0** | **91.4** | **91.4** | **Pi/SH/CH/051401/2012** |
| **28** | **84.9** | **86.4** | **87.5** | **72.0** | **85.0** | **86.6** | **86.6** | **84.4** | **87.4** | **84.9** | **85.3** | **83.6** | **83.5** | **85.5** | **71.6** | **91.8** | **92.4** | **93.5** | **94.9** | **94.9** | **94.9** | **99.8** | **94.9** | **99.0** | **99.7** | **99.8** | **99.9** |  | **99.8** | **99.8** | **99.0** | **92.0** | **91.4** | **91.4** | **Pi/SH/CH/051402/2012** |
| **29** | **85.1** | **86.5** | **87.7** | **72.0** | **85.3** | **86.8** | **86.8** | **84.6** | **87.6** | **85.2** | **85.6** | **83.6** | **83.7** | **85.7** | **71.6** | **92.0** | **92.6** | **93.8** | **95.1** | **95.1** | **95.1** | **100.0** | **95.2** | **99.2** | **99.9** | **99.8** | **99.7** | **99.8** |  | **100** | **99.1** | **92.1** | **91.6** | **91.6** | **Pi/SH/CH/061002/2011** |
| **30** | **85.0** | **86.5** | **87.6** | **72.0** | **85.2** | **86.6** | **86.7** | **84.6** | **87.5** | **85.0** | **85.5** | **83.5** | **83.5** | **85.6** | **71.7** | **91.9** | **92.5** | **93.6** | **95.0** | **95.0** | **95.0** | **99.9** | **95.0** | **99.1** | **99.8** | **99.8** | **99.7** | **99.8** | **99.9** |  | **99.1** | **92.1** | **91.6** | **91.6** | **Pi/SH/CH/120203/2011** |
| **31** | **84.9** | **85.8** | **87.0** | **72.3** | **85.2** | **85.9** | **86.2** | **84.6** | **87.0** | **85.0** | **85.2** | **83.0** | **83.3** | **85.3** | **72.2** | **91.1** | **91.7** | **92.6** | **94.1** | **94.1** | **94.1** | **98.6** | **94.2** | **98.0** | **98.7** | **984** | **98.4** | **98.4** | **98.7** | **98.7** |  | **91.8** | **91.3** | **91.4** | **pigeon/Ningxia/2068/2016** |
| **32** | **85.7** | **88.5** | **87.4** | **72.1** | **85.8** | **88.3** | **89.0** | **85.5** | **89.4** | **85.9** | **86.9** | **84.1** | **85.3** | **87.1** | **72.1** | **87.2** | **87.7** | **86.9** | **87.7** | **87.7** | **87.7** | **87.4** | **87.7** | **87.0** | **87.5** | **87.4** | **87.2** | **87.2** | **87.4** | **87.3** | **86.6** |  | **99.5** | **94.2** | **QH1** |
| **33** | **85.5** | **88.3** | **87.2** | **72.1** | **85.7** | **88.1** | **88.9** | **85.4** | **89.2** | **85.8** | **86.8** | **84.0** | **85.1** | **86.9** | **72.1** | **87.1** | **87.5** | **86.8** | **87.5** | **87.5** | **87.5** | **87.2** | **87.6** | **86.9** | **87.3** | **87.2** | **87.0** | **87.0** | **87.2** | **87.1** | **86.5** | **99.7** |  | **93.7** | **QH4** |
| **34** | **84.8** | **87.0** | **94.6** | **72.4** | **84.9** | **87.9** | **87.5** | **84.8** | **87.6** | **84.8** | **85.7** | **82.2** | **83.8** | **85.7** | **71.8** | **88.6** | **89.1** | **87.3** | **88.3** | **88.3** | **88.3** | **88.2** | **88.3** | **88.0** | **88.2** | **88.0** | **88.0** | **87.9** | **88.2** | **88.0** | **87.3** | **87.0** | **86.8** |  | **ZJ1** |

Note: Nucleic acid similarity was in the lower left corner；Amino acid similarity was in the upper right corner.
